# Real-Time Survey of Vaccine Safety of the mRNA-1273 SARS-CoV-2 Vaccine in Workplace Vaccination at Keio University

**DOI:** 10.3390/vaccines10091461

**Published:** 2022-09-03

**Authors:** Kaho Okumura, Azusa Hara, Isa Inada, Daisuke Sugiyama, Takahiro Hoshino, Takahiro Yakoh, Hirokazu Yokoyama, Hisashi Urushihara

**Affiliations:** 1 Division of Drug Development and Regulatory Science, Faculty of Pharmacy, Keio University, 1-5-30 Shibakoen, Minato-ku, Tokyo 105-8512, Japan; 2 Toshimagaoka Girls Senior High School, 1-25-22 Ikebukuro Toshima-ku, Tokyo 170-0013, Japan; 3 Faculty of Nursing and Medical Care, Keio University, 4411 Endoh, Fujisawa 252-0883, Japan; 4 Faculty of Economics, Keio University, 2-15-45 Mita, Minato-ku, Tokyo 108-8345, Japan; 5 Faculty of Science and Technology, Keio University, 3-14-1 Hiyoshi, Kohoku-ku, Yokohama 223-8522, Japan; 6 Health Center, Keio University, 2-15-45 Mita, Minato-ku, Tokyo 108-8345, Japan

**Keywords:** mRNA vaccines, vaccine safety, real world, real-time, Japan

## Abstract

The mRNA-1273 Moderna COVID-19 vaccine was introduced to combat the COVID-19 global pandemic in 2020. Although the safety of the vaccine has been investigated worldwide, real-world safety data is scarce in Japan. An online, real-time survey of adverse events following immunization (AEFIs) with mRNA-1273 was conducted in the setting of a workplace vaccination program at the School of Pharmacy, Keio University from 26 June 2021, to 11 June 2022. Participants were requested to take four surveys during a seven-day follow-up period after each of the first, second, and third booster doses. The maximum number of responses, from 301 respondents, was obtained on day 0 (vaccination date) for the first dose. 98% of respondents reported local and systemic AEFIs for the second dose on day 1. No noticeable difference in local reactions was seen among the three doses. Females reported more AEFIs than males, and the young group (18–29 years) reported a higher rate than the middle age group (≥30 years) after the first dose. Age and gender differences in rates decreased at the second and third doses. This survey confirmed that the safety profile of mRNA-1273 in a real-world setting was similar to that derived from the clinical trials, and that the agent was well-tolerated.

## 1. Background

Multiple COVID-19 vaccines have been developed at an unprecedented speed with the expectation that they will provide an effective countermeasure against the emerging threat to global public health of novel coronavirus infection [1]. Among these vaccines, the first two to become available, Pfizer-BioNTech BNT162b2 and Moderna mRNA-1273, use a new modality, termed lipid nanoparticle-encapsulated mRNA vaccine. This modality employs synthetic mRNAs which encode the spike protein of SARS-CoV-2 and is completely novel when compared to modalities already in wide use in conventional vaccines. A randomized, observer-blinded, placebo-controlled clinical trial of BNT162b2 COVID-19 mRNA-vaccine, with more than forty-three thousand participants, demonstrated vaccine effectiveness as high as 95%, enabling emergency use authorization (EUA) by the FDA on 11 December 2020, in the U.S. [2]. This was followed by a phase 3 trial of the mRNA-1273 COVID-19 vaccine, the COVE trial, which demonstrated vaccine effectiveness (94.1%) as high as that of the BNT162b2 vaccine, thereby also enabling EUA by FDA on 18 December 2020 [3].

The COVE trial to assess the safety and efficacy of mRNA-1273 revealed that solicited local adverse events following immunization (AEFIs), including injection site pain, erythema, induration, and tenderness, occurred more frequently in the mRNA-1273 group than in the placebo group and that the severity of these solicited systemic events increased after the second dose compared with the first. 

Several post-launch reports on vaccine safety for BNT162b2 and mRNA-1273 focused on safety in the acute phase after vaccination, including local reactions and anaphylaxis [4]. Based on safety monitoring conducted via the Vaccine Adverse Event Reporting System, the U.S. Centers for Disease Control and Prevention issued an alert concerning an increased risk of myocarditis and pericarditis among male adolescents and young adults administered mRNA vaccines, especially after the second dose [5]. 

Following EUA in the U.S. and U.K., the first special approval of BNT162b2 in Japan was obtained on 14 February 2021. Vaccination was prioritized for healthcare professionals and the elderly over the age of 65 [6,7]. Special approvals of mRNA-1273 in Japan were granted on 21 May 2021, after a domestic clinical trial showed seroconversion after vaccination in 147 healthy Japanese. However, this number of subjects was insufficient to determine the safety profile of mRNA-1273 [8]. A nationwide temporary vaccination program, including workplace vaccination, was initiated under Article 6 of the Immunization Act to accelerate the national vaccination uptake rate [9]. Upon implementation of the program, the government of Japan urged the population to undergo vaccination voluntarily but did not legally mandate vaccination. On 21 June 2021, following the implementation of government policy, a workplace vaccination program covering all people aged 20–65 years using mRNA-1273 was initiated at universities and major enterprises. The first wave of workplace vaccination at 1875 injection sites aimed to vaccinate more than 18 million individuals around Japan [10].

The Delta variant was the most prevalent circulating strain during the period of the first and second doses in the first wave of workplace vaccination programs, as was the Omicron variant at the time of the third booster dose in Japan [11]. The third-dose vaccine effectiveness of mRNA-1273 against Omicron infection in the US was reported to decrease to as low as 71.6%, compared with as high as 93.7% against Delta infection [12]. 

To date, no similar national-scale, wide-ranging vaccination program for young adults and the working general public has been undertaken in Japan, except for the regularly offered human papillomavirus vaccination for adolescent girls. Thus, continuous monitoring of the safety of these new vaccines is crucial [13]. Unfortunately, however, Japan does not have an immunization registry to enable national active surveillance for the tracking of all vaccinees to assess the efficacy and safety of new vaccines [14]. 

Upon implementation of workplace vaccination at Keio University, we conducted a real-time follow-up survey to assess AEFIs with mRNA-1273 in young- to middle-aged Japanese individuals, motivated by the lack of a national active surveillance system for vaccine safety in Japan.

## 2. Methods

### 2.1. Setting

Workplace vaccination at enterprises and universities was implemented beginning 21 June 2021, using the mRNA-1273 COVID-19 vaccine. The workplace vaccination program did not provide PCR/antigen tests before and after vaccination, nor did it entail serological tests to detect prior immunity.

Regarding the timing of administration, the first dose was initiated at the Tokyo Mita campus of Keio University on 21 June 2021; the second dose was finalized on 3 September 2021; and the third booster dose was given from 22 March 2022, to 31 March 2022, and on weekends between 21 May 2022 and 29 May 2022. All students, including undergraduates and graduates, and university staff and their families were targeted by email notification of the workplace vaccination program or by a posting on the Keio internet portal site, totaling more than thirty thousand subjects for the first and second dose. Among 1327 undergraduates and graduates at Keio University Faculty of Pharmacy, the first-dose vaccination rate was 85.8% (1138) and the two-dose rate was 85.0% (1128). The number of vaccinees who received the third dose under the workplace vaccination program at Keio University was small, at 6461, as vaccination programs for the third dose were initiated by municipalities beforehand, and as the third dose was given to as few as 61.8% of the Japanese population by 30 June 2022 [15]. 

This survey was approved by the Keio University Faculty of Pharmacy ethics review committee for medical and biological research involving human subjects on 21 June 2021 (No. 210621-1), and the protocol amendment was approved on 10 March 2022 (No. 220310-6).

### 2.2. Participants

Survey participants were recruited using posters placed around the campus of the School of Pharmacy, a post on the Keio internet portal site, and e-mail. Inclusion criteria were (1) receipt of at least one dose of mRNA-1273 vaccine as a workplace vaccination at Keio University, and (2) status as an undergraduate, graduate student, faculty, or administrative office staff member of the School of Pharmacy with a Keio ID number allowing access to the Keio intranet (keio.jp domain). All participants provided e-consent after reading an explanation of the intranet survey. Respondents who received doses outside of the workplace vaccination program at Keio University were excluded by an eligibility check item in the survey.

### 2.3. Questionnaire

An online, real-time survey of AEFIs was conducted during two periods. The first period was between 26 June 2021, and 14 September 2021, for the first and second dose, and the second period was between 22 March 2022, and 11 June 2022, for the third dose. Responses were collected via an anonymous, self-administered online questionnaire constructed using Google Forms on the Keio University intranet service and made accessible to the participants through their Keio ID (Box S1). Eight local (injection site pain, localized warmth, exanthema, erythema, swelling, itchiness, and red eye) and eleven systemic AEFIs (malaise, fever, fatigue, myalgia, arthralgia, nausea/vomiting, diarrhea, chills, headache, and rash) were enquired about with reference to the Japanese package insert of mRNA-1273 COVID-19 vaccine and the COVE trial. We added axillary swelling as a survey item for the third dose. Information on other unsolicited events (other local and/or systemic AEFIs); medical care-seeking behaviors, including doctor visits and use of antipyretic analgesics after vaccination; and background information (time of response, number of vaccination doses, date of vaccination, age, and sex) was also collected. Medical care-seeking behaviors were investigated as an objective surrogate of the severity of reported AEFIs. The questionnaire was administered on the day of vaccination (day 0), and one, three, and seven days after vaccination (day 1, 3, and 7, respectively) for each dose. Subjects were asked to answer the same questionnaire items four times during the one-week follow-up period after each dose of vaccination, making a total of 12 questionnaires for three doses. To maintain anonymity, responses were not annotated with the Keio ID, meaning that responses on one day could not be linked to those on another day in the same subject. Thus, none of the responses contained personally identifiable information.

### 2.4. Analysis

Study variables were summarized with proportions as percentages and were tabulated and illustrated for the whole population and by subgroups for each dose. Subgroup analyses by age (young group 18 to <30 years and middle-aged group ≥30 years) and sex were conducted. Between-group comparison was done using Fisher’s exact test; two-tailed with a significance level of 0.05. No adjustment for multiplicity was made due to safety endpoints. All responses were submitted for statistical analysis. 

## 3. Results

The number of respondents was 301 for day 0, 259 for day 1, 196 for day 3, and 143 for day 7 for the first dose; 203 for day 0, 179 for day 1, 138 for day 3, and 130 for day 7 for the second dose; and 42 for day 0, 38 for day 1, 35 for day 3, and 27 for day 7 for the third dose (Table 1). Approximately 60% of respondents were female, while approximately 70% of respondents were aged 18 to <30 years. The median time to response after vaccination ranged from 2.0 to 8.0 days for the first dose, from 4.0 to 12.5 days for the second, and from 2.0 to 9.0 days for the third. 

The number of respondents who reported any AEFI on day 0, day 1, day 3, and day 7 were 252 (83.7%), 235 (90.7%), 78 (39.8%), and 28 (19.6%), respectively, for the first dose. Respective numbers for the second dose were 188 (92.6%), 176 (98.3%), 73 (52.9%), and 14 (10.8%); and 36 (85.7%), 36 (94.7%), 16 (45.7%), and 3 (11.1%) for the third dose. The highest proportion of respondents reporting AEFIs was observed on day 1 for all doses. Reporting ratios of any AEFI were consistently higher for the second and third doses than for the first dose, except on day 7 (Table 2, Table 3 and Table 4). Overall, most AEFIs disappeared within seven days after vaccination for all doses. For local AEFIs, injection-site pain was a major event, with a reporting proportion of 78.7% for day 0, 87.6% for day 1, 30.1% for day 3, and 4.9% for day 7 for the first dose. Similar proportions were reported for the second and third doses. In contrast, other local AEFIs, including localized warmth, erythema, swelling, and itchiness, were reported more frequently for the second dose than the first. On day 7, among AEFIs, erythema (first vs. second dose, 5.6% vs. 0.8%), swelling (7.0% vs. 0%), and others (7.7% vs. 1.5%) were reported in small proportions but were more frequent with the first dose than the second. Among systemic AEFIs, malaise and fever were major AEFIs with reporting proportions of 15.6% and 13.3% for day 0, 38.2% and 30.9% for day 1, 8.2% and 5.6% for day 3, and 0.7% and 0% for day 7 for the first dose, respectively. Proportions of reported systemic AEFIs were prominently increased for the second dose compared to those for the first; for example, an approximately 60% increase for fever (first vs. second dose, 30.9% to 87.7%) and more than 30% for malaise (38.2% to 74.9%) on day 1. An increased number of responses was also seen for headaches, arthralgia, myalgia, chills, and fatigue. Reported proportions for the third dose were closely similar to those for the second dose. There were two cases of AEFI related to heart conditions, including palpitation in a woman in her twenties on day 1 of the second dose and mild chest pain in a man in his twenties on day 0 of the third dose, neither of whom sought medical care. In contrast to these high AEFI reporting rates, rates for doctor visits were substantially lower, with a maximum of 2.6% seen for day 1 with the third dose. Consistent with the high response rates for fever, use of antipyretic analgesics reached 71.5% on day 1 for the second dose and 55.3% on day 1 for the third dose, versus a maximum reported rate of 23.9% on day 1 for the first dose.

First-dose injection site pain was reported more commonly in females (85.5%) than in males (69.5%). Localized warmth and malaise were also reported more commonly in females (18.0% and 19.2%, respectively) than in males (6.3% and 10.2%, respectively) on day 0 (Figure S1 and Table S1). On day 1, injection site pain, localized warmth, swelling, malaise, fever, and headache were reported more commonly in females (94.3%, 25.5%, 22.9%, 45.9%, 38.2%, and 29.3%, respectively) than in males (77.5%, 13.7%, 9.8%, 26.5%, 19.6%, and 15.7%, respectively). Second-dose erythema was reported more commonly in females (12.9%) than in males (2.8%) on day 0 (Figure S2 and Table S1). On day 1, localized warmth, erythema, and swelling were also reported more commonly in females (55.4%, 18.8%, and 25.0%, respectively) than in males (38.8%, 4.5%, and 10.4%, respectively). On day 3, swelling was reported more commonly in females (18.1%) than in males (5.5%). For the third dose, however, the female subgroup reported similar rates to the male subgroup (Figure S3 and Table S1). 

First-dose localized warmth was reported more commonly in the young subgroup (15.1%) than in the middle-aged subgroup (4.8%) on day 0 (Figure S4 and Table S2). On day 1, injection site pain, localized warmth, malaise, fever, arthralgia, and headache were also reported more commonly in the young subgroup (90.5%, 25.1%, 41.7%, 35.7%, 13.6%, and 28.6%, respectively) than in the middle-aged subgroup (78.3%, 6.7%, 26.7%, 15.0%, 3.3%, and 8.3%, respectively). Only itchiness on day 3 for the first dose was higher (2.9% vs. 10.7%) in the middle-aged subgroup. Second-dose malaise and chills were reported more commonly in the young subgroup (57.3% and 30.1%, respectively) than in the middle-aged subgroup (38.3% and 13.3%, respectively) on day 0 (Figure S5 and Table S2). In contrast, the young subgroup reported similar rates for the third dose to the middle-aged subgroup, except for fatigue (30.0% vs. 0%) on day 0 (Figure S6 and Table S2).

## 4. Discussion

This analysis of self-reported, real-time surveillance monitoring of a workplace vaccination program for three doses included more than 500 doses of mRNA-1273 COVID-19 vaccine in a young- to middle-aged population in a university-based setting. Almost all respondents reported solicited AEFIs, probably arising from the immunogenicity of the mRNA vaccine, all of which appeared to resolve within a week after vaccination. Doctor visits after vaccination were rarely reported, likely reflecting a lack of serious complications requiring medical intervention shortly after vaccination.

### 4.1. Design and Relevance of the Present Survey

This survey was conducted as active surveillance of vaccine safety [13]. It complements knowledge gaps in mRNA vaccine safety in Japanese subjects present at a time when the evidence for vaccine safety was derived from only a small number of Japanese subjects tested in pre-authorization clinical trials before special approval and who were reported to have experienced suspected adverse reactions in a spontaneous reporting system in an unsolicited manner. Historically, post-launch vaccine safety in Japan has been assessed primarily by a reliance on passive surveillance methodologies. Despite the fact that nearly 80% of the Japanese population completed the primary vaccination with two doses within six months (April to October in 2020) as a result of workplace (including more than 19 million mRNA-1273 total doses) and regional vaccination programs (including more than 150 million BNT162b2 and 30 million mRNA-1273 total doses) [16], the number of people vaccinated under systematic active safety monitoring in Japan remains relatively small, due to the lack of a national immunization registry linked with outcome information stored in separate databases of electronic health records, which would aid scientifically valid pharmacoepidemiological assessment of vaccine efficacy and safety in a systematic way [14]. Governmental vaccine safety monitoring after launch was conducted by an expert vaccine safety assessment panel subcommittee within the Ministry of Health, Labor, and Welfare in Japan [16]. Its assessment has been very limited and has relied mainly on the analysis of safety information based on the spontaneous adverse event reporting system mentioned above, and immunization data based on the national Vaccine Record System (VRS) for individual vaccination records of COVID-19 vaccines [17]. VRS is not linkable with any medical records, including outcome information related to efficacy and safety at the individual level. In addition, the safety assessment of COVID-19 vaccines has also included the publicly funded cohort studies of the COVID-19 Vaccine Early Phase Intensive Survey program, as well as spontaneous reports [18]. The vaccine license holder of mRNA-1273 vaccine in Japan, Takeda Pharmaceuticals, is independently conducting post-marketing surveillance for long-term outcomes in efficacy and safety, including a questionnaire survey and cohort studies using a large-scale claims database, although the results have yet to be published in a scientific journal [8].

### 4.2. Comparison with Pre-Authorization Clinical Trials

A comparison of the first and second doses showed an overall increase in the frequency of all reported AEFIs for the second dose, as well as greater increases in the reporting rates of systemic AEFIs, including malaise, fever, fatigue myalgia, arthralgia, chills, and headache, (a net increase of 36.7%, 56.8%, 14.6%, 15.7%, 33.5%, 23.5%, and 30.3%, respectively). The same tendency was found in the phase I/II clinical trial conducted in 200 healthy Japanese adults by the marketing authorization holder, which reported a low rate of fever of 2.0% for the first dose and 42.9% for the second dose [8]. In the present survey, however, we found markedly higher reporting rates of fever for both the first and second doses, at 30.9% and 87.7%, respectively. Small but higher reporting rates of erythema and swelling on day 7 for the first dose than the second dose may be related to delayed localized cutaneous hypersensitivity reactions, the so-called “Moderna arm” that was also observed in the third dose [19].

Comparison of our present findings with the safety data of the COVE trial reveals several differences; our survey reported a constantly higher rate of fever (30.9% vs. 0.9% for first dose; 87.7% vs. 17.4% for second dose) and a lower rate of nausea/vomiting (3.5% vs. 9.4% for first dose; 6.7% vs. 21.4% for second dose) [3]. Interestingly, our present results overall resemble the self-reported rate of solicited adverse events among vaccinees of the COVE trial, even though there was a large difference in study design, particularly with regard to the COVE trial being a pre-marketing, well-controlled, randomized, observer-blinded, placebo-controlled design in which subjects were equipped with an electronic diary for AEFI reporting, and the voluntary nature of participation and reporting of AEFIs in the present survey. Our participants, all drawn from Keio University Faculty of Pharmacy, are likely to have been health-conscience and to have had a major interest in new vaccines and their AEFIs, which potentially contributed to the accuracy of reporting of the presence/absence of the solicited AEFIs, as shown by the results, which included many reports on the absence of AEFIs. The majority (more than 70%) of the study sample was comparably young, and this difference in age distribution may explain the remarkably high reporting rate of fever compared to all three of the preceding studies using the mRNA-1273 vaccine mentioned above, notwithstanding the voluntary reporting nature of AEFIs [3,8,18].

There were no major differences in reporting ratios between the second and third doses in the present survey. Results for the third dose in a phase IIa clinical trial of mRNA-1273 conducted in 344 American adults and a phase III clinical trial of mRNA-1273 conducted in 15,184 were consistent with the present survey [20].

### 4.3. Comparison with Other Real-World Findings

A real-world cohort study was conducted among thirteen thousand members of the Self-Defense Forces (SDF) who were vaccinated with mRNA-1273 immediately after its emergency approval by the COVID-19 Administrative Office of Juntendo University. This study—part of a publicly funded COVID-19 Vaccine Early Phase Intensive Survey—reported a self-reported rate of fever of as low as 7.09% for the first dose of mRNA-1273, compared with 30.9% in the present survey [18]. Comparison of these data may lack sufficient validity to draw any convincing inference because of differences in the characteristics of the SDF study population, which was dominated by male sex and healthier people. 

A self-report survey conducted at Okayama University, located in western Japan, reported a fever rate of 23.2% for the first dose and 88.0% for the second dose, similar to the present survey [21]. That survey was also conducted in the setting of a university-based workplace vaccination program using mRNA-1273. The subject characteristics and reporting rates were closely similar, suggesting the high validity of our survey results. The results of the above observational studies are summarized along with those of a systematic review of clinical trials in Table S3 [22].

Another study which evaluated the relation between fever intensity and antipyretic analgesic use in BNT162b2 mRNA vaccinees in a Japanese population reported fever rates as low as 60.4% after the second dose, compared with our present higher rate of 87.7% [23]. That study reported that maximum body temperature after the second dose was associated with IgG level. Our high reporting rate of fever may indicate a high immune response, particularly elicited among our young study population. Further, the reporting rates of fever in the two studies of the BNT162b2 vaccine were relatively lower than that observed in the present survey [23,24], and this observation may agree with the previous finding that mRNA-1273 vaccinees demonstrate a stronger immune response than BNT162b2 vaccinees [25].

### 4.4. Subgroup Analysis

The reporting proportions of any AEFI were mostly higher for females than males for the first dose, whereas the gap became smaller for the second dose. AEFI reporting rates in the young group (18 to <30 years) were higher than those of the middle-aged group (≥30 years) for the first dose. These gaps between age subgroups also became smaller for the second dose. Although female gender and young age were considered AEFI risk factors for the first dose, this cannot be said for the second dose.

### 4.5. Medical Care Sought

The use of antipyretic analgesics was higher for the second dose compared with the first dose, as the rate of fever increased for the second dose. The clinical trial of BNT162b2 also reported that the rate of use of antipyretic analgesics increased from the first to the second dose, but was nevertheless lower than our results, at 45%. This difference may be associated with the difference in the reporting rate of fever compared with the present study [2]. The significantly low rate of doctor visits indicates that there were no severe AEFIs in the short period immediately after vaccination. The COVID-19 vaccine appears well tolerated in our survey population, because almost no other serious outcomes such as myocarditis were observed.

## 5. Limitations

This study has several limitations. First, our respondents were not classified according to whether they had been infected with SARS-CoV2 preceding the vaccination or not; accordingly, the effects of a past history of SARS-CoV2 infection remain unknown, although this might have affected the occurrence of AEFIs [26,27]. Second, people who have experienced AEFIs may tend to respond and report AEFIs more because of the voluntary nature of responses, likely resulting in high reporting rates. Third respondents were not completely followed throughout the one-week follow-up period after immunization, and not all participants completed all four questionnaires during each follow-up period. In particular, in the latter part of each follow-up period (day 3 and day 7), fewer people responded to the questionnaire. The comparison validity among doses is therefore suboptimal due to the different composition of samples. Thus, the validity of the reporting ratios for the latter follow-up periods is questionable. Fourth, the grade of severity was indicated only by whether they had received medical care or had taken antipyretic analgesics, and additional data may therefore be needed for scrutiny. Fifth, even though many of the participants likely had basic knowledge of medicine, they may have misclassified AEFIs. Sixth, the number and distribution of participants were limited because of the university setting, and the survey results cannot therefore be generalized. Of approximately 1500 students and facility members of Keio University Faculty of Pharmacy, a maximum of 301 participants (approximately 20%) volunteered to participate. This real-time survey was designed to coincidentally start at the very beginning of the workplace vaccination program at Keio University, which likely helped minimize recall bias due to lack of coinstantaneity between the occurrence and recording of AEFIs. Seventh, the number of respondents for the third dose was too small for detailed analysis, because of the small number of participants in the workplace vaccination program at Keio University. 

## 6. Conclusions

This survey, conducted among a university-based workplace vaccination population, confirmed that the safety profile of mRNA-1273 COVID-19 vaccine in the Japanese working generation was similar to the safety profile observed in domestic and international pre-authorization clinical trials, despite its small sample size and voluntary participation-based design. High reporting rates of AEFIs in this young to middle-aged Japanese population were documented, particularly with regard to fever. All reported AEFIs could be considered as immune reactions after vaccination and were relatively mild. Establishment of an immunization registry with linkage to a monitoring system of important health outcomes, such as Vaccine Data Link, will aid in the conduct of high quality and valid research and surveillance to monitor vaccine efficacy and safety in Japan.

## Figures and Tables

**Table 1:** Demographic characteristics. IQR: interquartile range. Day 0 is defined as the day of immunization. Time to Response is defined as the number of days between immunization (day 0) and response time.

	Day 0		Day 1		Day 3		Day 7	
First Dose (n)	301		259		196		143	
Male (%)	128	(42.5%)	102	(39.4%)	80	(40.8%)	54	(37.8%)
Female (%)	172	(57.1%)	157	(60.6%)	116	(59.2%)	89	(62.2%)
18 to ≤29 yr (%)	238	(79.1%)	199	(76.8%)	140	(71.4%)	96	(67.1%)
30 to ≤69 yr (%)	63	(20.9%)	60	(23.2%)	56	(28.6%)	47	(32.9%)
Time to Response (IQR)	2.0	(1.0, 5.0)	2.0	(1.0, 5.0)	4.5	(3.0, 6.0)	8.0	(7.0, 10.0)
Second Dose (n)	203		179		138		130	
Male (%)	71	(35.0%)	67	(37.4%)	55	(39.9%)	47	(36.2%)
Female (%)	132	(65.0%)	112	(62.6%)	83	(60.1%)	83	(63.8%)
18 to ≤29 yr (%)	143	(70.4%)	122	(68.2%)	90	(65.2%)	84	(64.6%)
30 to ≤69 yr (%)	60	(29.6%)	57	(31.8%)	48	(34.8%)	46	(35.4%)
Time to Response (IQR)	7.0	(1.0, 19.0)	4.0	(2.0, 18.0)	9.0	(4.0, 20.0)	12.5	(8.0, 22.0)
Third Dose (n)	42		38		35		27	
Male (%)	16	(38.1%)	15	(39.5%)	14	(40.0%)	11	(40.7%)
Female (%)	26	(61.9%)	23	(60.5%)	21	(60.0%)	16	(59.3%)
18 to 29 yr (%)	30	(71.4%)	26	(68.4%)	24	(68.6%)	16	(59.3%)
30 to 69 yr (%)	12	(28.6%)	12	(31.6%)	11	(31.4%)	11	(40.7%)
Time to Response (IQR)	2.0	(1.0, 6.5)	3.0	(1.75, 8.0)	6.0	(3.0, 8.0)	9.0	(7.0, 12.0)

**Table 2:** Adverse events following immunization (AEFIs) for the first dose. AEFIs: adverse events following immunization. * Fisher’s exact test, significantly higher (*p* < 0.05) than the second dose (Table 3). † Other local AEFIs include numbness, sore throat, eye irritation, and others. ‡ Other systemic AEFIs include irregular bleeding, anorexia, cerebral anemia, and others. Day 0 is defined as the day of immunization.

		Day 0 (*n* = 301)		Day 1 (*n* = 259)		Day 3 (*n* = 196)		Day 7 (*n* = 143)	
**Any AEFI**		252	(83.7%)	235	(90.7%)	78	(39.8%)	28	(19.6%) *
Local	Injection-site pain	237	(78.7%)	227	(87.6%)	59	(30.1%)	7	(4.9%)
	Localized warmth	39	(13.0%)	54	(20.8%)	6	(3.1%)	3	(2.1%)
	Exanthema	2	(0.7%)	1	(0.4%)	0		1	(0.7%)
	Erythema	1	(0.3%)	3	(1.2%)	9	(4.6%)	8	(5.6%) *
	Swelling	30	(10.0%)	46	(17.8%)	10	(5.1%)	10	(7.0%) *
	Itchiness	0		6	(2.3%)	10	(5.1%)	11	(7.7%)
	Red eye	1	(0.3%)	1	(0.4%)	0		0	
	Others †	15	(5.0%)	15	(5.8%)	4	(2.0%)	11	(7.7%) *
Systemic	Malaise	47	(15.6%)	99	(38.2%)	16	(8.2%)	1	(0.7%)
	Fever	40	(13.3%)	80	(30.9%)	11	(5.6%)	0	
	Fatigue	29	(9.6%)	46	(17.8%)	7	(3.6%)	1	(0.7%)
	Myalgia	49	(16.3%)	59	(22.8%)	10	(5.1%)	0	
	Arthralgia	17	(5.6%)	29	(11.2%)	2	(1.0%)	1	(0.7%)
	Nausea/Vomiting	9	(3.0%)	9	(3.5%)	2	(1.0%)	0	
	Diarrhea	1	(0.3%)	10	(3.9%)	2	(1.0%)	0	
	Chills	6	(2.0%)	26	(10.0%)	2	(1.0%)	0	
	Headache	28	(9.3%)	62	(23.9%)	11	(5.6%)	4	(2.8%)
	Rash	0		1	(0.4%)	0		0	
	Others ‡	6	(2.0%)	3	(1.2%)	5	(2.6%)	0	
Medical Care Sought	Doctor visit	1	(0.3%)	0		1	(0.5%)	0	
	Use of analgesics	30	(10.0%)	62	(23.9%)	8	(4.1%)	2	(1.4%)

**Table 3:** Adverse events following immunization (AEFIs) for the second dose. AEFIs: adverse events following immunization. * Fisher’s exact test, significantly higher (*p* < 0.05) than the first dose (Table 2). † Other local AEFIs include nasal mucus, sore throat, and others. ‡ Other systemic AEFIs include dizziness, palpitation, sweating, and others. Day 0 is defined as the day of immunization.

		Day 0 (*n* = 203)		Day 1 (*n* = 179)		Day 3 (*n* = 138)		Day 7 (*n* = 130)	
**Any AEFI**		188	(92.6%) *	176	(98.3%) *	73	(52.9%) *	14	(10.8%)
Local	Injection-site pain	165	(81.3%)	154	(86.0%)	46	(33.3%)	3	(2.3%)
	Localized warmth	82	(40.4%) *	88	(49.2%) *	23	(16.7%) *	1	(0.8%)
	Exanthema	4	(2.0%)	3	(1.7%)	2	(1.4%)	1	(0.8%)
	Erythema	19	(9.4%) *	24	(13.4%) *	20	(14.5%) *	1	(0.8%)
	Swelling	26	(12.8%)	35	(19.6%)	18	(13.0%) *	0	
	Itchiness	12	(5.9%) *	12	(6.7%) *	28	(20.3%) *	5	(3.8%)
	Red eye	1	(0.5%)	1	(0.6%)	0		0	
	Others †	4	(2.0%)	5	(2.8%)	3	(2.2%)	2	(1.5%)
Systemic	Malaise	105	(51.7%) *	134	(74.9%) *	17	(12.3%)	5	(3.8%)
	Fever	108	(53.2%) *	157	(87.7%) *	8	(5.8%)	3	(2.3%)
	Fatigue	45	(22.2%) *	58	(32.4%) *	14	(10.1%) *	2	(1.5%)
	Myalgia	48	(23.6%)	69	(38.5%) *	9	(6.5%)	3	(2.3%)
	Arthralgia	37	(18.2%) *	80	(44.7%) *	4	(2.9%)	1	(0.8%)
	Nausea/Vomiting	7	(3.4%)	12	(6.7%)	3	(2.2%)	0	
	Diarrhea	3	(1.5%)	3	(1.7%)	1	(0.7%)	0	
	Chills	51	(25.1%) *	60	(33.5%) *	4	(2.9%)	0	
	Headache	59	(29.1%) *	97	(54.2%) *	32	(23.2%) *	5	(3.8%)
	Rash	1	(0.5%)	3	(1.7%)	1	(0.7%)	1	(0.8%)
	Others ‡	4	(2.0%)	13	(7.3%)	4	(2.9%)	0	
Medical Care Sought	Doctor visit	0		1	(0.6%)	1	(0.7%)	0	
	Use of analgesics	84	(41.4%) *	128	(71.5%) *	18	(13.0%) *	4	(3.1%)

**Table 4:** Adverse events following immunization (AEFIs) for the third dose. AEFIs: adverse events following immunization. † Fisher’s exact test, significantly higher (*p* < 0.05) than the first dose (Table 2). ‡ Other local AEFIs include sore neck, sore throat, and others. # Other systemic AEFIs include anorexia, hot flashes, mild chest pain, and others. Day 0 is defined as the day of immunization.

		Day 0 (*n* = 42)		Day 1 (*n* = 38)		Day 3 (*n* = 35)		Day 7 (*n* = 27)	
**Any AEFI**		36	(85.7%)	36	(94.7%)	16	(45.7%)	3	(11.1%)
Local	Injection-site pain	36	(85.7%)	36	(94.7%)	11	(31.4%)	1	(3.7%)
	Localized warmth	15	(35.7%) †	20	(52.6%) †	4	(11.4%) †	0	
	Exanthema	0		0		0		0	
	Erythema	0		1	(2.6%)	2	(5.7%)	0	
	Swelling	3	(7.1%)	4	(10.5%)	4	(11.4%)	0	
	Itchiness	0		0		4	(11.4%)	2	(7.4%)
	Red eye	0		1	(2.6%)	0		0	
	Axillary swelling	2	(4.8%)	5	(13.2%)	4	(11.4%)	1	(3.7%)
	Others ‡	1	(2.4%)	1	(2.6%)	0		2	(7.4%)
Systemic	Malaise	17	(40.5%) †	30	(78.9%) †	5	(14.3%)	0	
	Fever	12	(28.6%) †	31	(81.6%) †	1	(2.9%)	0	
	Fatigue	9	(21.4%) †	22	(57.9%) †	3	(8.6%)	0	
	Myalgia	6	(14.3%)	12	(31.6%)	2	(5.7%)	1	(3.7%)
	Arthralgia	9	(21.4%) †	14	(36.8%) †	1	(2.9%)	0	
	Nausea/Vomiting	1	(2.4%)	2	(5.3%)	0		0	
	Diarrhea	0		0		0		0	
	Chills	7	(16.7%) †	12	(31.6%) †	0		0	
	Headache	5	(11.9%)	17	(44.7%) †	0		0	
	Rash	0		0		0		0	
	Others #	2	(4.8%)	1	(2.6%)	0		0	
Medical Care Sought	Doctor visit	0		1	(2.6%)	0		0	
	Use of analgesics	12	(28.6%)	21	(55.3%)	2	(5.7%)	0	

## Data Availability

All data generated or analyzed during this study are included in this published article.

## Supplementary Materials

The following supporting information can be downloaded at: https://www.mdpi.com/article/10.3390/vaccines10091461/s1, Box S1: Survey items on AEFIs. Figure S1: Reporting Proportions of Solicited AEFIs by Sex for First Dose. Figure S2: Reporting Proportions of Solicited AEFIs by Sex for Second Dose. Figure S3: Reporting Proportions of Solicited AEFIs by Sex for Third Dose. Figure S4: Reporting Proportions of Solicited AEFIs by Age for First Dose. Figure S5: Reporting Proportions of Solicited AEFIs by Age for Second Dose. Figure S6: Reporting Proportions of Solicited AEFIs by Age for Third Dose. Table S1: *p*-values of Fisher’s Exact Tests by Gender Group Comparison. Table S2: *p*-values of Fisher’s Exact Tests by Age Group Comparison. Table S3: Comparison of reported rates in other observational studies and a systematic review of mRNA-1273 vaccine.

## List of Abbreviations

AEFIs:	adverse events following immunization
VRS:	vaccine record system
SDF:	self-defense forces
