# Supplementary material for: Real-Time Survey of Vaccine Safety of the mRNA-1273 SARS-CoV-2 Vaccine in Workplace Vaccination at Keio University
Source: Vaccines (Basel). 2022 Sep 3;10(9):1461. doi: 10.3390/vaccines10091461 (PMC9501947; doi:10.3390/vaccines10091461)
Supplement: Supplementary file 1 [file vaccines-10-01461-s001.zip › Supplemental Box220825.pdf]

Box S1: Survey items on AEFIs

1. Do you belong to Keio University Faculty of Pharmacy?
   - Yes
   - No
2. Is this a response about vaccination at the workplace program at Keio University?
   - Yes
   - No
3. Is this a response about the first or second or third dose? (depending on timing)
   - Yes
   - No
4. Date of vaccination
5. Age
   - Teens
   - Twenties
   - Thirties
   - Forties
   - Fifties
   - Sixties
6. Gender
   - Female
   - Male
   - Do not want to disclose
7. Do you have any side effects from the vaccine?
   - Yes
   - No
8. Please choose all applicable kind of side effects:
   Local
   - Injection-site pain
   - Localized warmth
   - Exanthema
   - Erythema
   - Swelling
   - Itchiness
   - Red eye
   - Axillary swelling (only for third dose)

- Others (When selected, please provide details of symptoms)

Systemic

- Malaise
- Fever
- Fatigue
- Myalgia
- Arthralgia
- Nausea/Vomiting
- Diarrhea
- Chills
- Headache
- Rash
- Others (When selected, please provide details of symptoms)

9. Did you visit any hospital or clinic because of the vaccine's side effects?
    - Yes
    - No
10. What medicines did you use for the vaccine's side effects?
    - None
    - Analgesics
    - Antiallergic drug
    - Others
    - Not sure what kind of medicines
