# Supplementary material for: Real-Time Survey of Vaccine Safety of the mRNA-1273 SARS-CoV-2 Vaccine in Workplace Vaccination at Keio University
Source: Vaccines (Basel). 2022 Sep 3;10(9):1461. doi: 10.3390/vaccines10091461 (PMC9501947; doi:10.3390/vaccines10091461)
Supplement: Supplementary file 1 [file vaccines-10-01461-s001.zip › Supplemental Tables220902.pdf]

Table S1. P-values of Fisher's Exact Tests by gender group comparison

		Day 0			Day 1			Day 3			Day 7		
Dose		First	Second	Third	First	Second	Third	First	Second	Third	First	Second	Third
Any side effect		.005	.159	.658	<.001	.557	1.000	.882	.084	.166	.004	.018	1.000
Local	Injection-site pain	.001	.851	.658	<.001	.121	1.000	.874	.140	.137	.254	.553	1.000
	Localized warmth	.003	.456	.746	.028	.044	.320	1.000	1.000	.635	1.000	1.000	-
	Exanthema	1.000	.613	-	1.000	1.000	-	-	.517	-	1.000	1.000	-
	Erythema	1.000	.022	-	.281	.006	1.000	.085	.216	1.000	.259	1.000	-
	Swelling	.079	.194	1.000	.008	.020	1.000	.532	.039	.635	.060	1.000	-
	Itchiness	-	1.000	-	1.000	.215	-	.050	.086	.635	.208	.159	.499
	Red eye	.427	1.000	-	1.000	1.000	1.000	-	-	-	-	-	-
Systemic	Malaise	.036	.660	.518	.002	.859	1.000	.797	.189	.627	1.000-	.653	-
	Fever	.390	.303	.158	.002	.815	.681	.761	.713	1.000	-	.553	-
	Fatigue	1.000	.861	.711	.511	.870	.506	1.000	.161	1.000	1.000	.535	-
	Myalgia	.750	.119	1.000	.763	.268	1.000	1.000	1.000	.506	1.000	1.000	1.000
	Arthralgia	.440	.182	.711	.688	.538	1.000	1.000	1.000	1.000	-	1.000	-
	Nausea/Vomiting	.737	1.000	1.000	.490	.215	.509	1.000	.276	-	-		-
	Diarrhea	.427	1.000	-	1.000	.051	-	.165	1.000	-	-	-	-
	Chills	1.000	.311	.690	.207	.418	1.000	.514	1.000	-	-	-	-
	Headache	.684	.422	.633	.017	.216	.744	.530	.306	-	.633	.159	-
	Rash	-	.350	-	1.000	1.000	-	-	1.000	-	-	1.000	-

Table S2. P-values of Fisher's Exact Tests by age group comparison

Young group 18 to <30 yr vs. middle-aged group ≥30 yr

		Day 0			Day 1			Day 3			Day 7		
Dose		First	Second	Third	First	Second	Third	First	Second	Third	First	Second	Third
Any side effect		.565	.384	.655	.039	.552	1.000	.421	.376	.273	1.000	.563	.056
Local	Injection-site pain	.227	.435	.655	.023	.103	1.000	.732	.455	.709	1.000	.552	.407
	Localized warmth	.033	.349	.726	.002	.630	1.000	.676	1.000	1.000	1.000	1.000	-
	Exanthema	1.000	1.000	-	1.000	.552	-	-	.543	-	1.000	1.000	-
	Erythema	.209	.598	-	.135	1.000	.316	.717	.135	1.000	.718	.354	-
	Swelling	.477	.822	1.000	1.000	.840	.577	.475	.294	.575	.166	.354	-
	Itchiness	-	.343	-	.625	.524	-	.034	.183	.082	.178	.053	.157
	Red eye	1.000	1.000	-	1.000	1.000	1.000	-		-	-	-	-
Systemic	Malaise	.847	.014	.300	.048	1.000	.393	1.000	1.000	.297	1.000	.345	-
	Fever	.093	.090	.128	.002	1.000	1.000	.184	.713	.314	-	.552	-
	Fatigue	.227	.063	.041	.084	.304	.725	.675	.770	1.000	1.000	.123	-
	Myalgia	.705	.474	.159	.863	.410	.268	1.000	.496	1.000	-	.285	.407
	Arthralgia	1.000	.320	.247	.033	.632	.296	1.000	1.000	.314	.329	1.000	-
	Nausea/Vomiting	.212	.424	1.000	.689	.755	.094	1.000	1.000	-	-	-	-
	Diarrhea	1.000	.557	-	1.000	1.000	-	.081	.348	-	-	-	-
	Chills	.350	.013	.651	.807	.737	.268	1.000	1.000	-	-	-	-
	Headache	.223	.090	.298	<.001	.260	.486	.732	.405	-	1.000	.655	-
	Rash	-	.296	-	1.000	1.000	-	-	1.000	-	-	1.000	-

Table S3. Comparison of reported rates in other observational studies and a meta-analysis of mRNA-1273 vaccine.
AEFIs with rates <1% are not listed for other studies.

* The rates of the present study indicate the maximum rates reported among three doses.

† The rates of meta-analysis indicate the minimum and maximum rates in mRNA-1273 vaccine clinical trials.

[1] Ito S. Interim analysis of health diary (17), COVID-19 Vaccine Early Phase Intensive Survey (cohort study) (in Japanese). The 73rd Immunization and Vaccine Committee Adverse reaction Assessment Subcommittee, R3 23rd Pharmaceutical Affairs Committee Drug Safety Countermeasure Subcommittee, Tokyo, Japan, 3 December 2021.

[2] Okayama university. The final report: a survey of adverse reaction of the mRNA-1273 SARS-CoV-2 Vaccine (in Japanese). Available online: https://www.okayama-u.ac.jp/up_load_files/press_r3/press20210915.pdf (accessed on 27 June 2022).

[3] McDonald I, Murray SM, Reynolds CJ, Altmann DM, Boyton RJ. Comparative systematic review and meta-analysis of reactogenicity, immunogenicity and efficacy of vaccines against SARS-CoV-2. *Npj Vaccines* **2021**, *6,* https://doi.org/10.1038/s41541-021-00336-1.

		First dose				Second dose			
		Present survey (max) *	Juntendo [1] N=12,578	Okayama [2] N=3,917	Meta-analysis [3] (min, max) †	Present survey (max) *	Juntendo [1] N=11,791	Okayama [2] N=3,288	Meta-analysis [3] (min, max) †
Local	Injection-site pain	87.6%	84.6%	91.4 %	62.5%, 86.7%	86.0%	84.2%	90.0%	75.0%, 91.7%
	Localized warmth	20.8%	10.9%			49.2%	32.1%		
	Exanthema	0.7%				2.0%			
	Erythema	5.6%	9.8%	19.7%		14.5%	25.7%	40.6%	
	Swelling	17.8%	10.3%	40.1%	4%, 10.7%	19.6%	20.1%	55.3%	8.5%, 12.2%
	Itchiness	7.7%	5.2%	17.5%		20.3%	13.9%	31.5%	
	Red eye	0.4%				0.6%			
Systemic	Malaise	38.2%	25.7%			74.9%	80.4%		
	Fever	30.9%	7.1%	23.2%	0%, 0.8%	87.7%	77.0%	88.0%	7.5%, 32.0%
	Fatigue	17.8%		48.4%	24.5%, 37.2%	32.4%		84.2%	50%, 65.3%
	Myalgia	22.8%		59.3%	12.5%, 22.7%	38.5%	1.7%	64.4%	47.5%, 58%
	Arthralgia	11.2%		15.9%	6.7%, 16.6%	44.7%	6.1%	50.2%	12.5%, 42.8%
	Nausea/Vomiting	3.5%		5.0%	0%, 8.3%	6.7%		12.7%	17%, 24.0%
	Diarrhea	3.9%		4.4%		1.7%		8.4%	
	Chills	10.0%		12.6%	6%, 8.3%	33.5%	5.5%	63.0%	32%, 59.3%
	Headache	23.9%	16.4%	29.6%	12.5%, 32.7%	54.2%	64.1%	74.7%	40%, 60.7%
	Rash	0.4%		2.4%		1.7%		5.7%	
