# Supplementary material for: Real-Time Survey of Vaccine Safety of the mRNA-1273 SARS-CoV-2 Vaccine in Workplace Vaccination at Keio University
Source: Vaccines (Basel). 2022 Sep 3;10(9):1461. doi: 10.3390/vaccines10091461 (PMC9501947; doi:10.3390/vaccines10091461)
Supplement: Supplementary file 1 [file vaccines-10-01461-s001.zip › Suppplemental Figure220902.pptx]

## Slide 1
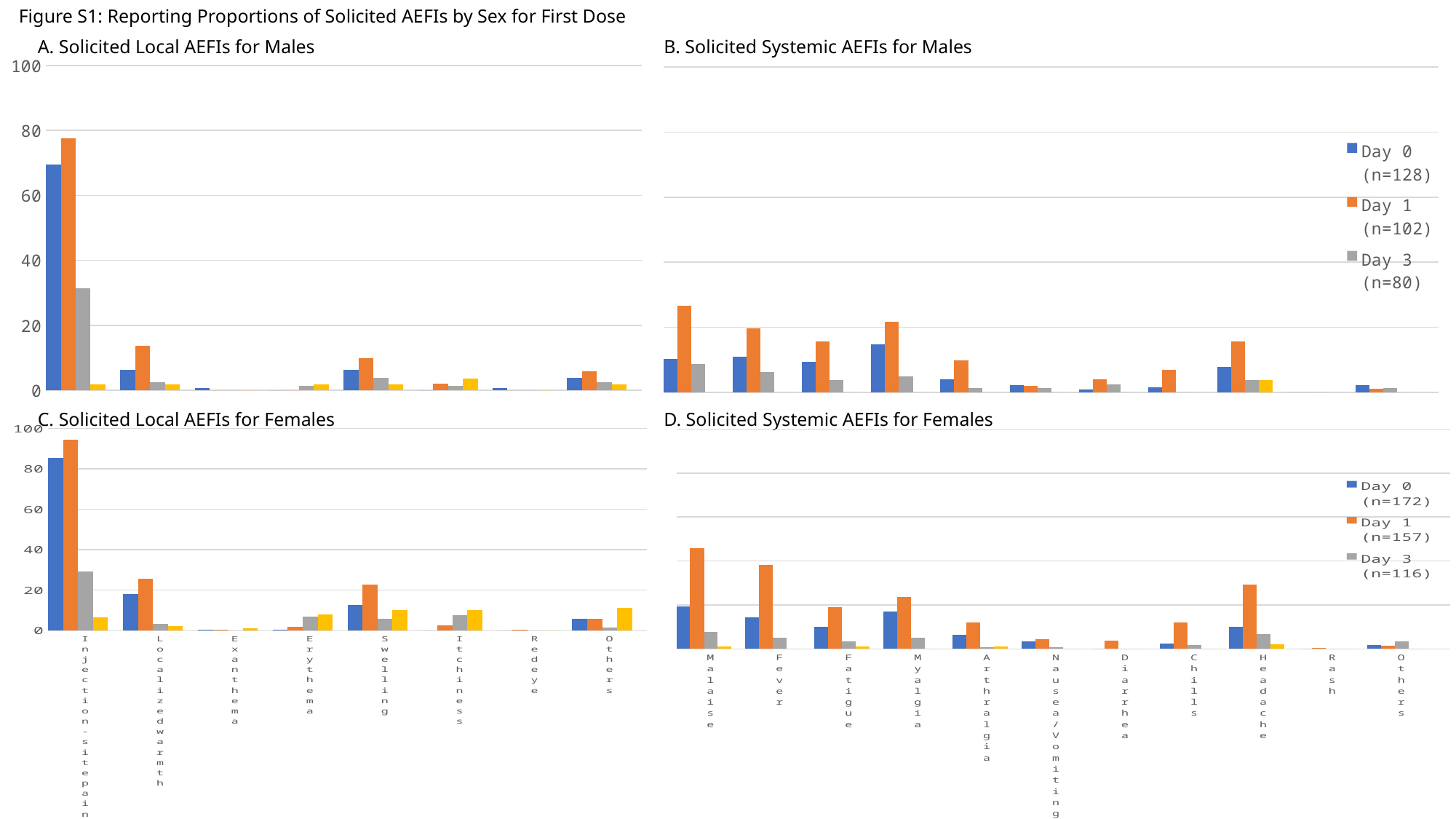

Figure S1: Reporting Proportions of Solicited AEFIs by Sex for First Dose
A. Solicited Local AEFIs for Males
B. Solicited Systemic AEFIs for Males
### Chart
| Category | Day 0 | Day 1 | Day 3 | Day 7 |
|---|---|---|---|---|
| | 69.5 | None | None | None |
| | None | 77.5 | None | None |
| Injection-site pain | None | None | 31.3 | None |
| | None | None | None | 1.9 |
| | None | None | None | None |
| | 6.3 | None | None | None |
| | None | 13.700000000000001 | None | None |
| Localized warmth | None | None | 2.5 | None |
| | None | None | None | 1.9 |
| | None | None | None | None |
| | 0.8 | None | None | None |
| | None | 0.0 | None | None |
| Exanthema | None | None | 0.0 | None |
| | None | None | None | 0.0 |
| | None | None | None | None |
| | 0.0 | None | None | None |
| | None | 0.0 | None | None |
| Erythema | None | None | 1.3 | None |
| | None | None | None | 1.9 |
| | None | None | None | None |
| | 6.3 | None | None | None |
| | None | 9.8 | None | None |
| Swelling | None | None | 3.8 | None |
| | None | None | None | 1.9 |
| | None | None | None | None |
| | 0.0 | None | None | None |
| | None | 2.0 | None | None |
| Itchiness | None | None | 1.3 | None |
| | None | None | None | 3.6999999999999997 |
| | None | None | None | None |
| | 0.8 | None | None | None |
| | None | 0.0 | None | None |
| Red eye | None | None | 0.0 | None |
| | None | None | None | 0.0 |
| | None | None | None | None |
| | 3.9 | None | None | None |
| | None | 5.8999999999999995 | None | None |
| Others | None | None | 2.5 | None |
| | None | None | None | 1.9 |
| | None | None | None | None |
### Chart
| Category | Day 0 | Day 1 | Day 3 | Day 7 |
|---|---|---|---|---|
| | 10.2 | None | None | None |
| | None | 26.5 | None | None |
| Malaise | None | None | 8.799999999999999 | None |
| | None | None | None | 0.0 |
| | None | None | None | None |
| | 10.9 | None | None | None |
| | None | 19.6 | None | None |
| Fever | None | None | 6.3 | None |
| | None | None | None | 0.0 |
| | None | None | None | None |
| | 9.4 | None | None | None |
| | None | 15.7 | None | None |
| Fatigue | None | None | 3.8 | None |
| | None | None | None | 0.0 |
| | None | None | None | None |
| | 14.799999999999999 | None | None | None |
| | None | 21.6 | None | None |
| Myalgia | None | None | 5.0 | None |
| | None | None | None | 0.0 |
| | None | None | None | None |
| | 3.9 | None | None | None |
| | None | 9.8 | None | None |
| Arthralgia | None | None | 1.3 | None |
| | None | None | None | 0.0 |
| | None | None | None | None |
| | 2.3 | None | None | None |
| | None | 2.0 | None | None |
| Nausea/
Vomiting | None | None | 1.3 | None |
| | None | None | None | 0.0 |
| | None | None | None | None |
| | 0.8 | None | None | None |
| | None | 3.9 | None | None |
| Diarrhea | None | None | 2.5 | None |
| | None | None | None | 0.0 |
| | None | None | None | None |
| | 1.6 | None | None | None |
| | None | 6.9 | None | None |
| Chills | None | None | 0.0 | None |
| | None | None | None | 0.0 |
| | None | None | None | None |
| | 7.8 | None | None | None |
| | None | 15.7 | None | None |
| Headache | None | None | 3.8 | None |
| | None | None | None | 3.6999999999999997 |
| | None | None | None | None |
| | 0.0 | None | None | None |
| | None | 0.0 | None | None |
| Rash | None | None | 0.0 | None |
| | None | None | None | 0.0 |
| | None | None | None | None |
| | 2.3 | None | None | None |
| | None | 1.0 | None | None |
| Others | None | None | 1.3 | None |
| | None | None | None | 0.0 |
| | None | None | None | None |
| | None | None | None | None |C. Solicited Local AEFIs for Females
D. Solicited Systemic AEFIs for Females
### Chart
| Category | Day 0 | Day 1 | Day 3 | Day 7 |
|---|---|---|---|---|
| | 85.5 | None | None | None |
| | None | 94.3 | None | None |
| Injection-site pain | None | None | 29.299999999999997 | None |
| | None | None | None | 6.7 |
| | None | None | None | None |
| | 18.0 | None | None | None |
| | None | 25.5 | None | None |
| Localized warmth | None | None | 3.4000000000000004 | None |
| | None | None | None | 2.1999999999999997 |
| | None | None | None | None |
| | 0.6 | None | None | None |
| | None | 0.6 | None | None |
| Exanthema | None | None | 0.0 | None |
| | None | None | None | 1.0999999999999999 |
| | None | None | None | None |
| | 0.6 | None | None | None |
| | None | 1.9 | None | None |
| Erythema | None | None | 6.9 | None |
| | None | None | None | 7.9 |
| | None | None | None | None |
| | 12.8 | None | None | None |
| | None | 22.900000000000002 | None | None |
| Swelling | None | None | 6.0 | None |
| | None | None | None | 10.100000000000001 |
| | None | None | None | None |
| | 0.0 | None | None | None |
| | None | 2.5 | None | None |
| Itchiness | None | None | 7.8 | None |
| | None | None | None | 10.100000000000001 |
| | None | None | None | None |
| | 0.0 | None | None | None |
| | None | 0.6 | None | None |
| Red eye | None | None | 0.0 | None |
| | None | None | None | 0.0 |
| | None | None | None | None |
| | 5.800000000000001 | None | None | None |
| | None | 5.7 | None | None |
| Others | None | None | 1.7000000000000002 | None |
| | None | None | None | 11.200000000000001 |
| | None | None | None | None |
### Chart
| Category | Day 0 | Day 1 | Day 3 | Day 7 |
|---|---|---|---|---|
| | 19.2 | None | None | None |
| | None | 45.9 | None | None |
| Malaise | None | None | 7.8 | None |
| | None | None | None | 1.0999999999999999 |
| | None | None | None | None |
| | 14.499999999999998 | None | None | None |
| | None | 38.2 | None | None |
| Fever | None | None | 5.2 | None |
| | None | None | None | 0.0 |
| | None | None | None | None |
| | 9.9 | None | None | None |
| | None | 19.1 | None | None |
| Fatigue | None | None | 3.4000000000000004 | None |
| | None | None | None | 1.0999999999999999 |
| | None | None | None | None |
| | 16.900000000000002 | None | None | None |
| | None | 23.599999999999998 | None | None |
| Myalgia | None | None | 5.2 | None |
| | None | None | None | 0.0 |
| | None | None | None | None |
| | 6.4 | None | None | None |
| | None | 12.1 | None | None |
| Arthralgia | None | None | 0.8999999999999999 | None |
| | None | None | None | 1.0999999999999999 |
| | None | None | None | None |
| | 3.5000000000000004 | None | None | None |
| | None | 4.5 | None | None |
| Nausea/
Vomiting | None | None | 0.8999999999999999 | None |
| | None | None | None | 0.0 |
| | None | None | None | None |
| | 0.0 | None | None | None |
| | None | 3.8 | None | None |
| Diarrhea | None | None | 0.0 | None |
| | None | None | None | 0.0 |
| | None | None | None | None |
| | 2.3 | None | None | None |
| | None | 12.1 | None | None |
| Chills | None | None | 1.7000000000000002 | None |
| | None | None | None | 0.0 |
| | None | None | None | None |
| | 9.9 | None | None | None |
| | None | 29.299999999999997 | None | None |
| Headache | None | None | 6.9 | None |
| | None | None | None | 2.1999999999999997 |
| | None | None | None | None |
| | 0.0 | None | None | None |
| | None | 0.6 | None | None |
| Rash | None | None | 0.0 | None |
| | None | None | None | 0.0 |
| | None | None | None | None |
| | 1.7000000000000002 | None | None | None |
| | None | 1.3 | None | None |
| Others | None | None | 3.4000000000000004 | None |
| | None | None | None | 0.0 |
| | None | None | None | None |
| | None | None | None | None |

## Slide 2
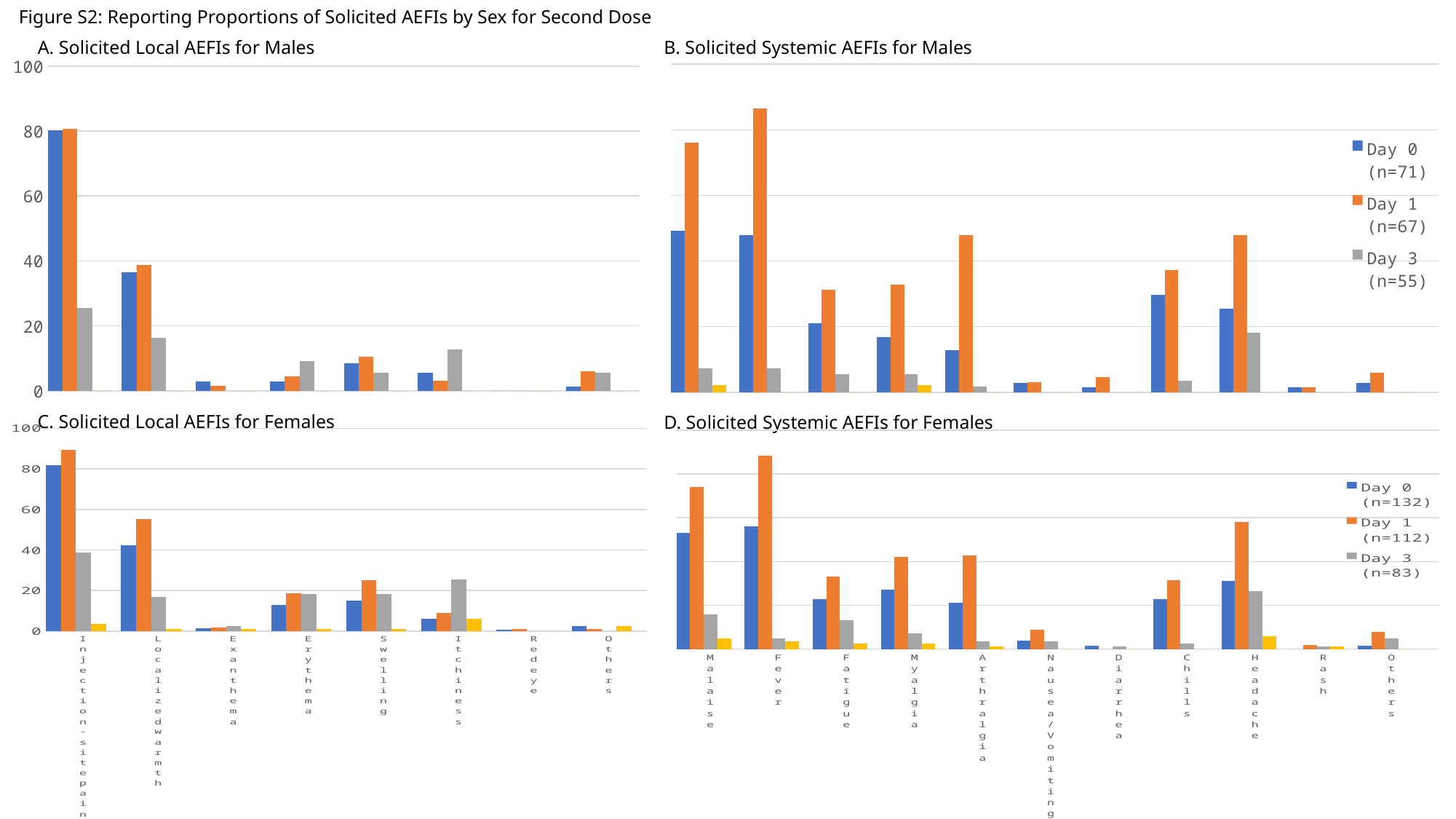

Figure S2: Reporting Proportions of Solicited AEFIs by Sex for Second Dose
A. Solicited Local AEFIs for Males
B. Solicited Systemic AEFIs for Males
### Chart
| Category | Day 0 | Day 1 | Day 3 | Day 7 |
|---|---|---|---|---|
| | 80.30000000000001 | None | None | None |
| | None | 80.60000000000001 | None | None |
| Injection-site pain | None | None | 25.5 | None |
| | None | None | None | 0.0 |
| | None | None | None | None |
| | 36.6 | None | None | None |
| | None | 38.800000000000004 | None | None |
| Localized warmth | None | None | 16.400000000000002 | None |
| | None | None | None | 0.0 |
| | None | None | None | None |
| | 2.8000000000000003 | None | None | None |
| | None | 1.5 | None | None |
| Exanthema | None | None | 0.0 | None |
| | None | None | None | 0.0 |
| | None | None | None | None |
| | 2.8000000000000003 | None | None | None |
| | None | 4.5 | None | None |
| Erythema | None | None | 9.1 | None |
| | None | None | None | 0.0 |
| | None | None | None | None |
| | 8.5 | None | None | None |
| | None | 10.4 | None | None |
| Swelling | None | None | 5.5 | None |
| | None | None | None | 0.0 |
| | None | None | None | None |
| | 5.6000000000000005 | None | None | None |
| | None | 3.0 | None | None |
| Itchiness | None | None | 12.7 | None |
| | None | None | None | 0.0 |
| | None | None | None | None |
| | 0.0 | None | None | None |
| | None | 0.0 | None | None |
| Red eye | None | None | 0.0 | None |
| | None | None | None | 0.0 |
| | None | None | None | None |
| | 1.4000000000000001 | None | None | None |
| | None | 6.0 | None | None |
| Others | None | None | 5.5 | None |
| | None | None | None | 0.0 |
| | None | None | None | None |
### Chart
| Category | Day 0 | Day 1 | Day 3 | Day 7 |
|---|---|---|---|---|
| | 49.3 | None | None | None |
| | None | 76.1 | None | None |
| Malaise | None | None | 7.3 | None |
| | None | None | None | 2.1 |
| | None | None | None | None |
| | 47.9 | None | None | None |
| | None | 86.6 | None | None |
| Fever | None | None | 7.3 | None |
| | None | None | None | 0.0 |
| | None | None | None | None |
| | 21.099999999999998 | None | None | None |
| | None | 31.3 | None | None |
| Fatigue | None | None | 5.5 | None |
| | None | None | None | 0.0 |
| | None | None | None | None |
| | 16.900000000000002 | None | None | None |
| | None | 32.800000000000004 | None | None |
| Myalgia | None | None | 5.5 | None |
| | None | None | None | 2.1 |
| | None | None | None | None |
| | 12.7 | None | None | None |
| | None | 47.8 | None | None |
| Arthralgia | None | None | 1.7999999999999998 | None |
| | None | None | None | 0.0 |
| | None | None | None | None |
| | 2.8000000000000003 | None | None | None |
| | None | 3.0 | None | None |
| Nausea/
Vomiting | None | None | 0.0 | None |
| | None | None | None | 0.0 |
| | None | None | None | None |
| | 1.4000000000000001 | None | None | None |
| | None | 4.5 | None | None |
| Diarrhea | None | None | 0.0 | None |
| | None | None | None | 0.0 |
| | None | None | None | None |
| | 29.599999999999998 | None | None | None |
| | None | 37.3 | None | None |
| Chills | None | None | 3.5999999999999996 | None |
| | None | None | None | 0.0 |
| | None | None | None | None |
| | 25.4 | None | None | None |
| | None | 47.8 | None | None |
| Headache | None | None | 18.2 | None |
| | None | None | None | 0.0 |
| | None | None | None | None |
| | 1.4000000000000001 | None | None | None |
| | None | 1.5 | None | None |
| Rash | None | None | 0.0 | None |
| | None | None | None | 0.0 |
| | None | None | None | None |
| | 2.8000000000000003 | None | None | None |
| | None | 6.0 | None | None |
| Others | None | None | 0.0 | None |
| | None | None | None | 0.0 |
| | None | None | None | None |
| | None | None | None | None |C. Solicited Local AEFIs for Females
D. Solicited Systemic AEFIs for Females
### Chart
| Category | Day 0 | Day 1 | Day 3 | Day 7 |
|---|---|---|---|---|
| | 53.0 | None | None | None |
| | None | 74.1 | None | None |
| Malaise | None | None | 15.7 | None |
| | None | None | None | 4.8 |
| | None | None | None | None |
| | 56.10000000000001 | None | None | None |
| | None | 88.4 | None | None |
| Fever | None | None | 4.8 | None |
| | None | None | None | 3.5999999999999996 |
| | None | None | None | None |
| | 22.7 | None | None | None |
| | None | 33.0 | None | None |
| Fatigue | None | None | 13.3 | None |
| | None | None | None | 2.4 |
| | None | None | None | None |
| | 27.3 | None | None | None |
| | None | 42.0 | None | None |
| Myalgia | None | None | 7.199999999999999 | None |
| | None | None | None | 2.4 |
| | None | None | None | None |
| | 21.2 | None | None | None |
| | None | 42.9 | None | None |
| Arthralgia | None | None | 3.5999999999999996 | None |
| | None | None | None | 1.2 |
| | None | None | None | None |
| | 3.8 | None | None | None |
| | None | 8.9 | None | None |
| Nausea/
Vomiting | None | None | 3.5999999999999996 | None |
| | None | None | None | 0.0 |
| | None | None | None | None |
| | 1.5 | None | None | None |
| | None | 0.0 | None | None |
| Diarrhea | None | None | 1.2 | None |
| | None | None | None | 0.0 |
| | None | None | None | None |
| | 22.7 | None | None | None |
| | None | 31.3 | None | None |
| Chills | None | None | 2.4 | None |
| | None | None | None | 0.0 |
| | None | None | None | None |
| | 31.1 | None | None | None |
| | None | 57.99999999999999 | None | None |
| Headache | None | None | 26.5 | None |
| | None | None | None | 6.0 |
| | None | None | None | None |
| | 0.0 | None | None | None |
| | None | 1.7999999999999998 | None | None |
| Rash | None | None | 1.2 | None |
| | None | None | None | 1.2 |
| | None | None | None | None |
| | 1.5 | None | None | None |
| | None | 8.0 | None | None |
| Others | None | None | 4.8 | None |
| | None | None | None | 0.0 |
| | None | None | None | None |
| | None | None | None | None |
### Chart
| Category | Day 0 | Day 1 | Day 3 | Day 7 |
|---|---|---|---|---|
| | 81.8 | None | None | None |
| | None | 89.3 | None | None |
| Injection-site pain | None | None | 38.6 | None |
| | None | None | None | 3.5999999999999996 |
| | None | None | None | None |
| | 42.4 | None | None | None |
| | None | 55.400000000000006 | None | None |
| Localized warmth | None | None | 16.900000000000002 | None |
| | None | None | None | 1.2 |
| | None | None | None | None |
| | 1.5 | None | None | None |
| | None | 1.7999999999999998 | None | None |
| Exanthema | None | None | 2.4 | None |
| | None | None | None | 1.2 |
| | None | None | None | None |
| | 12.9 | None | None | None |
| | None | 18.8 | None | None |
| Erythema | None | None | 18.099999999999998 | None |
| | None | None | None | 1.2 |
| | None | None | None | None |
| | 15.2 | None | None | None |
| | None | 25.0 | None | None |
| Swelling | None | None | 18.099999999999998 | None |
| | None | None | None | 1.2 |
| | None | None | None | None |
| | 6.1 | None | None | None |
| | None | 8.9 | None | None |
| Itchiness | None | None | 25.3 | None |
| | None | None | None | 6.0 |
| | None | None | None | None |
| | 0.8 | None | None | None |
| | None | 0.8999999999999999 | None | None |
| Red eye | None | None | 0.0 | None |
| | None | None | None | 0.0 |
| | None | None | None | None |
| | 2.3 | None | None | None |
| | None | 0.8999999999999999 | None | None |
| Others | None | None | 0.0 | None |
| | None | None | None | 2.4 |
| | None | None | None | None |

## Slide 3
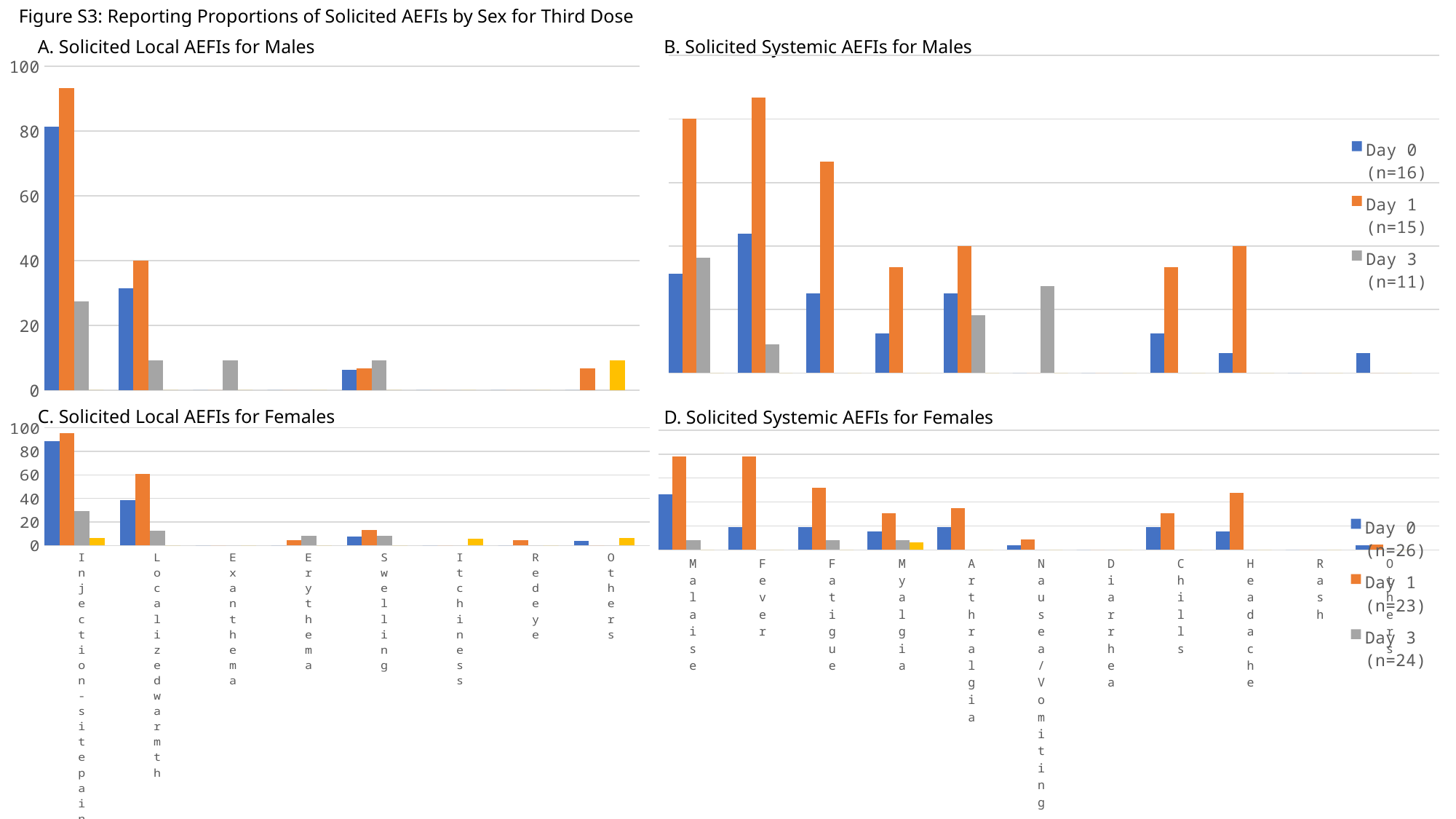

Figure S3: Reporting Proportions of Solicited AEFIs by Sex for Third Dose
A. Solicited Local AEFIs for Males
B. Solicited Systemic AEFIs for Males
### Chart
| Category | Day 0 | Day 1 | Day 3 | Day 7 |
|---|---|---|---|---|
| | 81.3 | None | None | None |
| | None | 93.3 | None | None |
| Injection-site pain | None | None | 27.3 | None |
| | None | None | None | 0.0 |
| | None | None | None | None |
| | 31.3 | None | None | None |
| | None | 40.0 | None | None |
| Localized warmth | None | None | 9.1 | None |
| | None | None | None | 0.0 |
| | None | None | None | None |
| | 0.0 | None | None | None |
| | None | 0.0 | None | None |
| Exanthema | None | None | 9.1 | None |
| | None | None | None | 0.0 |
| | None | None | None | None |
| | 0.0 | None | None | None |
| | None | 0.0 | None | None |
| Erythema | None | None | 0.0 | None |
| | None | None | None | 0.0 |
| | None | None | None | None |
| | 6.3 | None | None | None |
| | None | 6.7 | None | None |
| Swelling | None | None | 9.1 | None |
| | None | None | None | 0.0 |
| | None | None | None | None |
| | 0.0 | None | None | None |
| | None | 0.0 | None | None |
| Itchiness | None | None | 0.0 | None |
| | None | None | None | 0.0 |
| | None | None | None | None |
| | 0.0 | None | None | None |
| | None | 0.0 | None | None |
| Red eye | None | None | 0.0 | None |
| | None | None | None | 0.0 |
| | None | None | None | None |
| | 0.0 | None | None | None |
| | None | 6.7 | None | None |
| Others | None | None | 0.0 | None |
| | None | None | None | 9.1 |
| | None | None | None | None |
### Chart
| Category | Day 0 | Day 1 | Day 3 | Day 7 |
|---|---|---|---|---|
| | 31.3 | None | None | None |
| | None | 80.0 | None | None |
| Malaise | None | None | 36.4 | None |
| | None | None | None | 0.0 |
| | None | None | None | None |
| | 43.8 | None | None | None |
| | None | 86.7 | None | None |
| Fever | None | None | 9.1 | None |
| | None | None | None | 0.0 |
| | None | None | None | None |
| | 25.0 | None | None | None |
| | None | 66.7 | None | None |
| Fatigue | None | None | 0.0 | None |
| | None | None | None | 0.0 |
| | None | None | None | None |
| | 12.5 | None | None | None |
| | None | 33.3 | None | None |
| Myalgia | None | None | 0.0 | None |
| | None | None | None | 0.0 |
| | None | None | None | None |
| | 25.0 | None | None | None |
| | None | 40.0 | None | None |
| Arthralgia | None | None | 18.2 | None |
| | None | None | None | 0.0 |
| | None | None | None | None |
| | 0.0 | None | None | None |
| | None | 0.0 | None | None |
| Nausea/
Vomiting | None | None | 27.3 | None |
| | None | None | None | 0.0 |
| | None | None | None | None |
| | 0.0 | None | None | None |
| | None | 0.0 | None | None |
| Diarrhea | None | None | 0.0 | None |
| | None | None | None | 0.0 |
| | None | None | None | None |
| | 12.5 | None | None | None |
| | None | 33.3 | None | None |
| Chills | None | None | 0.0 | None |
| | None | None | None | 0.0 |
| | None | None | None | None |
| | 6.3 | None | None | None |
| | None | 40.0 | None | None |
| Headache | None | None | 0.0 | None |
| | None | None | None | 0.0 |
| | None | None | None | None |
| | 0.0 | None | None | None |
| | None | 0.0 | None | None |
| Rash | None | None | 0.0 | None |
| | None | None | None | 0.0 |
| | None | None | None | None |
| | 6.3 | None | None | None |
| | None | 0.0 | None | None |
| Others | None | None | 0.0 | None |
| | None | None | None | 0.0 |
| | None | None | None | None |
| | None | None | None | None |C. Solicited Local AEFIs for Females
D. Solicited Systemic AEFIs for Females
### Chart
| Category | Day 0 | Day 1 | Day 3 | Day 7 |
|---|---|---|---|---|
| | 88.5 | None | None | None |
| | None | 95.7 | None | None |
| Injection-site pain | None | None | 29.2 | None |
| | None | None | None | 6.3 |
| | None | None | None | None |
| | 38.5 | None | None | None |
| | None | 60.9 | None | None |
| Localized warmth | None | None | 12.5 | None |
| | None | None | None | 0.0 |
| | None | None | None | None |
| | 0.0 | None | None | None |
| | None | 0.0 | None | None |
| Exanthema | None | None | 0.0 | None |
| | None | None | None | 0.0 |
| | None | None | None | None |
| | 0.0 | None | None | None |
| | None | 4.3 | None | None |
| Erythema | None | None | 8.3 | None |
| | None | None | None | 0.0 |
| | None | None | None | None |
| | 7.7 | None | None | None |
| | None | 13.0 | None | None |
| Swelling | None | None | 8.3 | None |
| | None | None | None | 0.0 |
| | None | None | None | None |
| | 0.0 | None | None | None |
| | None | 0.0 | None | None |
| Itchiness | None | None | 0.0 | None |
| | None | None | None | 6.0 |
| | None | None | None | None |
| | 0.0 | None | None | None |
| | None | 4.3 | None | None |
| Red eye | None | None | 0.0 | None |
| | None | None | None | 0.0 |
| | None | None | None | None |
| | 3.8 | None | None | None |
| | None | 0.0 | None | None |
| Others | None | None | 0.0 | None |
| | None | None | None | 6.3 |
| | None | None | None | None |
### Chart
| Category | Day 0 | Day 1 | Day 3 | Day 7 |
|---|---|---|---|---|
| | 46.2 | None | None | None |
| | None | 78.3 | None | None |
| Malaise | None | None | 8.3 | None |
| | None | None | None | 0.0 |
| | None | None | None | None |
| | 19.2 | None | None | None |
| | None | 78.3 | None | None |
| Fever | None | None | 0.0 | None |
| | None | None | None | 0.0 |
| | None | None | None | None |
| | 19.2 | None | None | None |
| | None | 52.2 | None | None |
| Fatigue | None | None | 8.3 | None |
| | None | None | None | 0.0 |
| | None | None | None | None |
| | 15.4 | None | None | None |
| | None | 30.4 | None | None |
| Myalgia | None | None | 8.3 | None |
| | None | None | None | 6.3 |
| | None | None | None | None |
| | 19.2 | None | None | None |
| | None | 34.8 | None | None |
| Arthralgia | None | None | 0.0 | None |
| | None | None | None | 0.0 |
| | None | None | None | None |
| | 3.8 | None | None | None |
| | None | 8.7 | None | None |
| Nausea/
Vomiting | None | None | 0.0 | None |
| | None | None | None | 0.0 |
| | None | None | None | None |
| | 0.0 | None | None | None |
| | None | 0.0 | None | None |
| Diarrhea | None | None | 0.0 | None |
| | None | None | None | 0.0 |
| | None | None | None | None |
| | 19.2 | None | None | None |
| | None | 30.4 | None | None |
| Chills | None | None | 0.0 | None |
| | None | None | None | 0.0 |
| | None | None | None | None |
| | 15.4 | None | None | None |
| | None | 47.8 | None | None |
| Headache | None | None | 0.0 | None |
| | None | None | None | 0.0 |
| | None | None | None | None |
| | 0.0 | None | None | None |
| | None | 0.0 | None | None |
| Rash | None | None | 0.0 | None |
| | None | None | None | 0.0 |
| | None | None | None | None |
| | 3.8 | None | None | None |
| | None | 4.3 | None | None |
| Others | None | None | 0.0 | None |
| | None | None | None | 0.0 |
| | None | None | None | None |
| | None | None | None | None |

## Slide 4
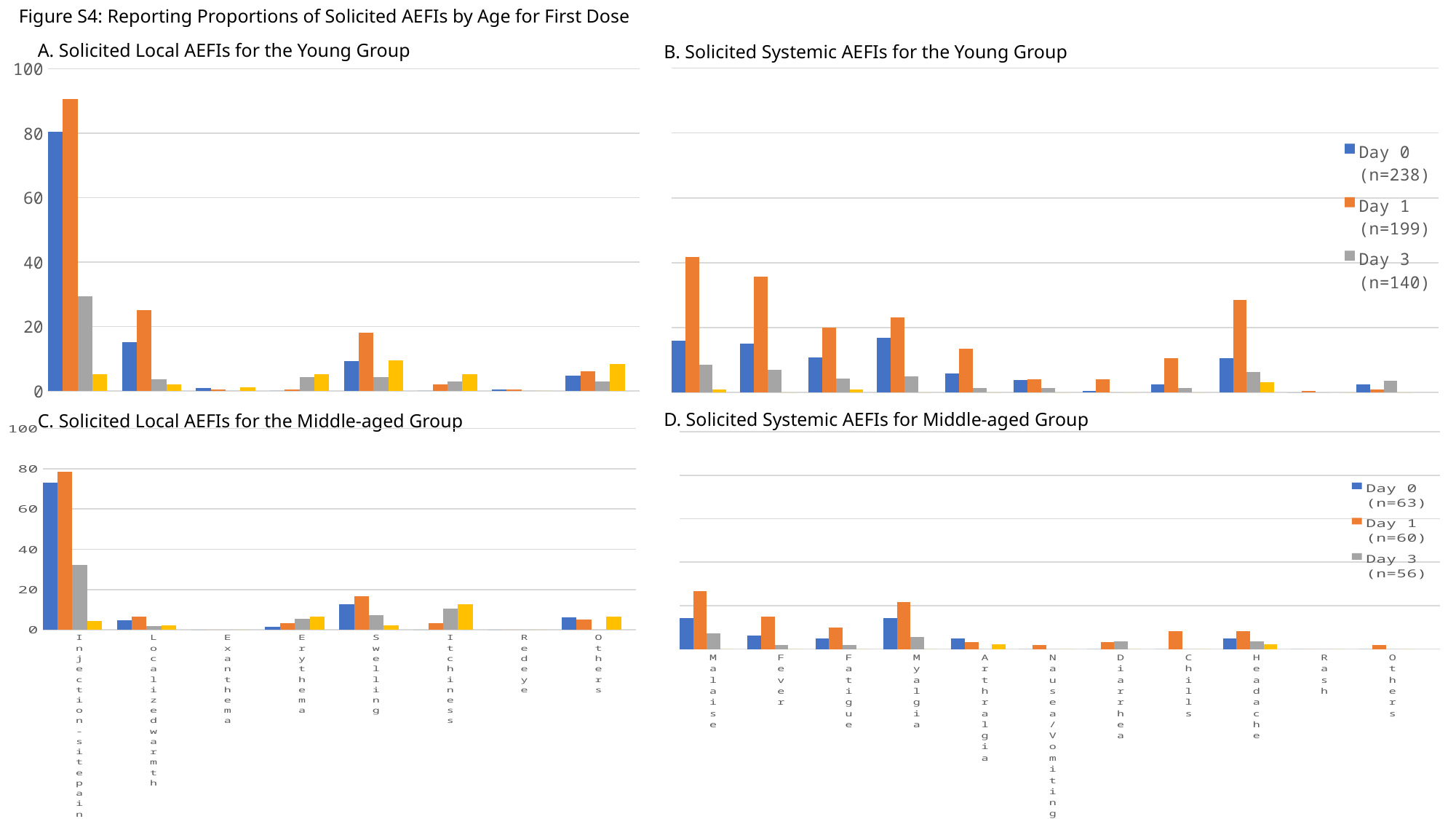

Figure S4: Reporting Proportions of Solicited AEFIs by Age for First Dose
A. Solicited Local AEFIs for the Young Group
B. Solicited Systemic AEFIs for the Young Group
### Chart
| Category | Day 0 | Day 1 | Day 3 | Day 7 |
|---|---|---|---|---|
| | 80.30000000000001 | None | None | None |
| | None | 90.5 | None | None |
| Injection-site pain | None | None | 29.299999999999997 | None |
| | None | None | None | 5.2 |
| | None | None | None | None |
| | 15.1 | None | None | None |
| | None | 25.1 | None | None |
| Localized warmth | None | None | 3.5999999999999996 | None |
| | None | None | None | 2.1 |
| | None | None | None | None |
| | 0.8 | None | None | None |
| | None | 0.5 | None | None |
| Exanthema | None | None | 0.0 | None |
| | None | None | None | 1.0 |
| | None | None | None | None |
| | 0.0 | None | None | None |
| | None | 0.5 | None | None |
| Erythema | None | None | 4.3 | None |
| | None | None | None | 5.2 |
| | None | None | None | None |
| | 9.2 | None | None | None |
| | None | 18.099999999999998 | None | None |
| Swelling | None | None | 4.3 | None |
| | None | None | None | 9.4 |
| | None | None | None | None |
| | 0.0 | None | None | None |
| | None | 2.0 | None | None |
| Itchiness | None | None | 2.9000000000000004 | None |
| | None | None | None | 5.2 |
| | None | None | None | None |
| | 0.4 | None | None | None |
| | None | 0.5 | None | None |
| Red eye | None | None | 0.0 | None |
| | None | None | None | 0.0 |
| | None | None | None | None |
| | 4.6 | None | None | None |
| | None | 6.0 | None | None |
| Others | None | None | 2.9000000000000004 | None |
| | None | None | None | 8.3 |
| | None | None | None | None |
### Chart
| Category | Day 0 | Day 1 | Day 3 | Day 7 |
|---|---|---|---|---|
| | 16.0 | None | None | None |
| | None | 41.699999999999996 | None | None |
| Malaise | None | None | 8.6 | None |
| | None | None | None | 1.0 |
| | None | None | None | None |
| | 15.1 | None | None | None |
| | None | 35.699999999999996 | None | None |
| Fever | None | None | 7.1 | None |
| | None | None | None | 0.0 |
| | None | None | None | None |
| | 10.9 | None | None | None |
| | None | 20.1 | None | None |
| Fatigue | None | None | 4.3 | None |
| | None | None | None | 1.0 |
| | None | None | None | None |
| | 16.8 | None | None | None |
| | None | 23.1 | None | None |
| Myalgia | None | None | 5.0 | None |
| | None | None | None | 0.0 |
| | None | None | None | None |
| | 5.8999999999999995 | None | None | None |
| | None | 13.600000000000001 | None | None |
| Arthralgia | None | None | 1.4000000000000001 | None |
| | None | None | None | 0.0 |
| | None | None | None | None |
| | 3.8 | None | None | None |
| | None | 4.0 | None | None |
| Nausea/
Vomiting | None | None | 1.4000000000000001 | None |
| | None | None | None | 0.0 |
| | None | None | None | None |
| | 0.4 | None | None | None |
| | None | 4.0 | None | None |
| Diarrhea | None | None | 0.0 | None |
| | None | None | None | 0.0 |
| | None | None | None | None |
| | 2.5 | None | None | None |
| | None | 10.6 | None | None |
| Chills | None | None | 1.4000000000000001 | None |
| | None | None | None | 0.0 |
| | None | None | None | None |
| | 10.5 | None | None | None |
| | None | 28.599999999999998 | None | None |
| Headache | None | None | 6.4 | None |
| | None | None | None | 3.1 |
| | None | None | None | None |
| | 0.0 | None | None | None |
| | None | 0.5 | None | None |
| Rash | None | None | 0.0 | None |
| | None | None | None | None |
| | None | None | None | 0.0 |
| | 2.5 | None | None | None |
| | None | 1.0 | None | None |
| Others | None | None | 3.5999999999999996 | None |
| | None | None | None | 0.0 |
| | None | None | None | None |
| | None | None | None | None |D. Solicited Systemic AEFIs for Middle-aged Group
C. Solicited Local AEFIs for the Middle-aged Group
### Chart
| Category | Day 0 | Day 1 | Day 3 | Day 7 |
|---|---|---|---|---|
| | 73.0 | None | None | None |
| | None | 78.3 | None | None |
| Injection-site pain | None | None | 32.1 | None |
| | None | None | None | 4.3 |
| | None | None | None | None |
| | 4.8 | None | None | None |
| | None | 6.7 | None | None |
| Localized warmth | None | None | 1.7999999999999998 | None |
| | None | None | None | 2.1 |
| | None | None | None | None |
| | 0.0 | None | None | None |
| | None | 0.0 | None | None |
| Exanthema | None | None | 0.0 | None |
| | None | None | None | 0.0 |
| | None | None | None | None |
| | 1.6 | None | None | None |
| | None | 3.3000000000000003 | None | None |
| Erythema | None | None | 5.4 | None |
| | None | None | None | 6.4 |
| | None | None | None | None |
| | 12.7 | None | None | None |
| | None | 16.7 | None | None |
| Swelling | None | None | 7.1 | None |
| | None | None | None | 2.1 |
| | None | None | None | None |
| | 0.0 | None | None | None |
| | None | 3.3000000000000003 | None | None |
| Itchiness | None | None | 10.7 | None |
| | None | None | None | 12.8 |
| | None | None | None | None |
| | 0.0 | None | None | None |
| | None | 0.0 | None | None |
| Red eye | None | None | 0.0 | None |
| | None | None | None | 0.0 |
| | None | None | None | None |
| | 6.3 | None | None | None |
| | None | 5.0 | None | None |
| Others | None | None | 0.0 | None |
| | None | None | None | 6.4 |
| | None | None | None | None |
### Chart
| Category | Day 0 | Day 1 | Day 3 | Day 7 |
|---|---|---|---|---|
| | 14.299999999999999 | None | None | None |
| | None | 26.700000000000003 | None | None |
| Malaise | None | None | 7.1 | None |
| | None | None | None | 0.0 |
| | None | None | None | None |
| | 6.3 | None | None | None |
| | None | 15.0 | None | None |
| Fever | None | None | 1.7999999999999998 | None |
| | None | None | None | 0.0 |
| | None | None | None | None |
| | 4.8 | None | None | None |
| | None | 10.0 | None | None |
| Fatigue | None | None | 1.7999999999999998 | None |
| | None | None | None | 0.0 |
| | None | None | None | None |
| | 14.299999999999999 | None | None | None |
| | None | 21.7 | None | None |
| Myalgia | None | None | 5.4 | None |
| | None | None | None | 0.0 |
| | None | None | None | None |
| | 4.8 | None | None | None |
| | None | 3.3000000000000003 | None | None |
| Arthralgia | None | None | 0.0 | None |
| | None | None | None | 2.1 |
| | None | None | None | None |
| | 0.0 | None | None | None |
| | None | 1.7000000000000002 | None | None |
| Nausea/
Vomiting | None | None | 0.0 | None |
| | None | None | None | 0.0 |
| | None | None | None | None |
| | 0.0 | None | None | None |
| | None | 3.3000000000000003 | None | None |
| Diarrhea | None | None | 3.5999999999999996 | None |
| | None | None | None | 0.0 |
| | None | None | None | None |
| | 0.0 | None | None | None |
| | None | 8.3 | None | None |
| Chills | None | None | 0.0 | None |
| | None | None | None | 0.0 |
| | None | None | None | None |
| | 4.8 | None | None | None |
| | None | 8.3 | None | None |
| Headache | None | None | 3.5999999999999996 | None |
| | None | None | None | 2.1 |
| | None | None | None | None |
| | 0.0 | None | None | None |
| | None | 0.0 | None | None |
| Rash | None | None | 0.0 | None |
| | None | None | None | 0.0 |
| | None | None | None | None |
| | 0.0 | None | None | None |
| | None | 1.7000000000000002 | None | None |
| Others | None | None | 0.0 | None |
| | None | None | None | 0.0 |
| | None | None | None | None |
| | None | None | None | None |

## Slide 5
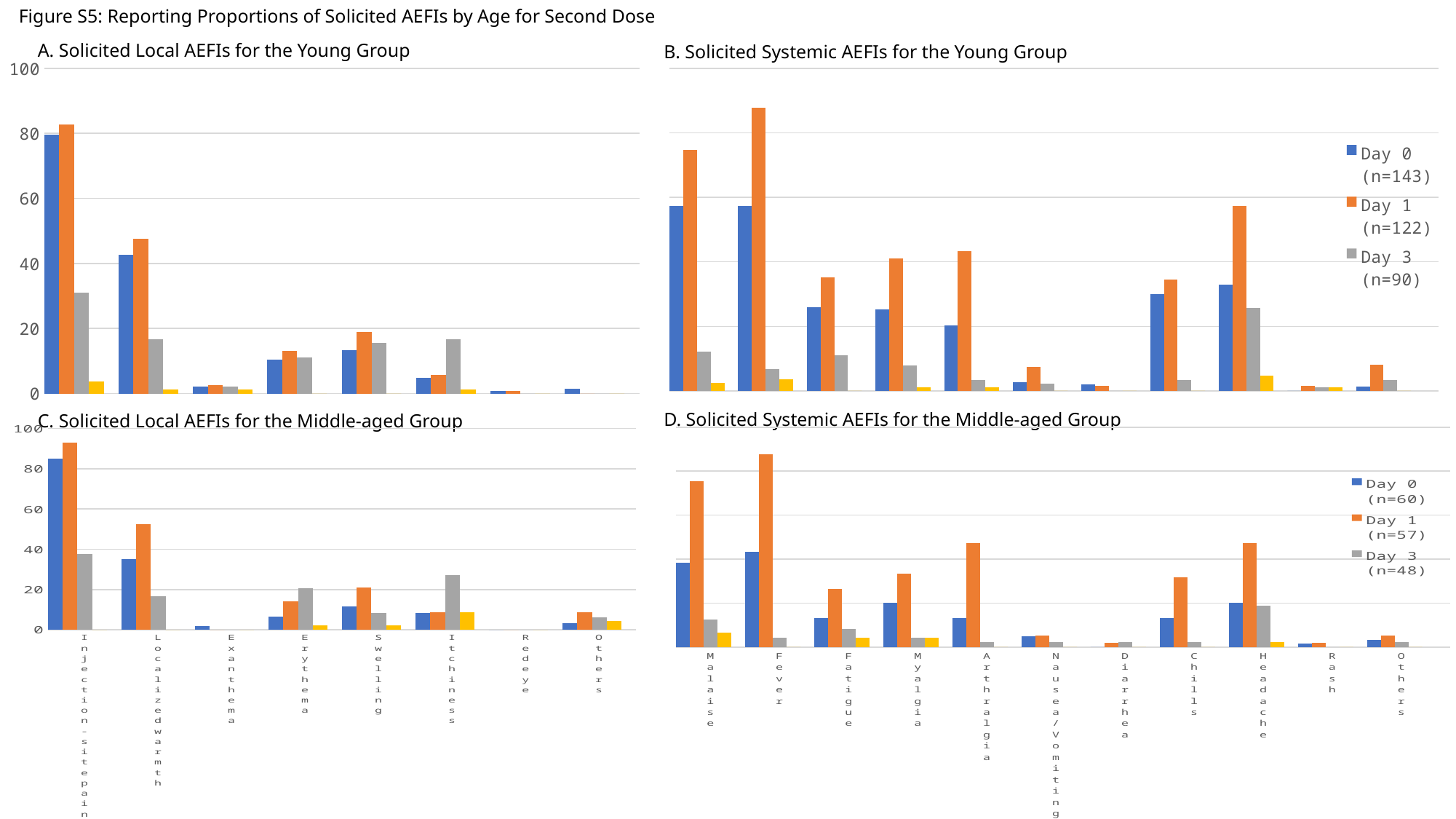

Figure S5: Reporting Proportions of Solicited AEFIs by Age for Second Dose
A. Solicited Local AEFIs for the Young Group
B. Solicited Systemic AEFIs for the Young Group
### Chart
| Category | Day 0 | Day 1 | Day 3 | Day 7 |
|---|---|---|---|---|
| | 79.7 | None | None | None |
| | None | 82.8 | None | None |
| Injection-site pain | None | None | 31.1 | None |
| | None | None | None | 3.6 |
| | None | None | None | None |
| | 42.7 | None | None | None |
| | None | 47.5 | None | None |
| Localized warmth | None | None | 16.7 | None |
| | None | None | None | 1.2 |
| | None | None | None | None |
| | 2.1 | None | None | None |
| | None | 2.5 | None | None |
| Exanthema | None | None | 2.2 | None |
| | None | None | None | 1.2 |
| | None | None | None | None |
| | 10.5 | None | None | None |
| | None | 13.1 | None | None |
| Erythema | None | None | 11.1 | None |
| | None | None | None | 0.0 |
| | None | None | None | None |
| | 13.3 | None | None | None |
| | None | 18.9 | None | None |
| Swelling | None | None | 15.6 | None |
| | None | None | None | 0.0 |
| | None | None | None | None |
| | 4.9 | None | None | None |
| | None | 5.7 | None | None |
| Itchiness | None | None | 16.7 | None |
| | None | None | None | 1.2 |
| | None | None | None | None |
| | 0.7 | None | None | None |
| | None | 0.8 | None | None |
| Red eye | None | None | 0.0 | None |
| | None | None | None | 0.0 |
| | None | None | None | None |
| | 1.4 | None | None | None |
| | None | 0.0 | None | None |
| Others | None | None | 0.0 | None |
| | None | None | None | 0.0 |
| | None | None | None | None |
### Chart
| Category | Day 0 | Day 1 | Day 3 | Day 7 |
|---|---|---|---|---|
| | 57.3 | None | None | None |
| | None | 74.6 | None | None |
| Malaise | None | None | 12.2 | None |
| | None | None | None | 2.4 |
| | None | None | None | None |
| | 57.3 | None | None | None |
| | None | 87.7 | None | None |
| Fever | None | None | 6.7 | None |
| | None | None | None | 3.6 |
| | None | None | None | None |
| | 25.9 | None | None | None |
| | None | 35.2 | None | None |
| Fatigue | None | None | 11.1 | None |
| | None | None | None | 0.0 |
| | None | None | None | None |
| | 25.2 | None | None | None |
| | None | 41.0 | None | None |
| Myalgia | None | None | 7.8 | None |
| | None | None | None | 1.2 |
| | None | None | None | None |
| | 20.3 | None | None | None |
| | None | 43.4 | None | None |
| Arthralgia | None | None | 3.3 | None |
| | None | None | None | 1.2 |
| | None | None | None | None |
| | 2.8 | None | None | None |
| | None | 7.4 | None | None |
| Nausea/
Vomiting | None | None | 2.2 | None |
| | None | None | None | 0.0 |
| | None | None | None | None |
| | 2.1 | None | None | None |
| | None | 1.6 | None | None |
| Diarrhea | None | None | 0.0 | None |
| | None | None | None | 0.0 |
| | None | None | None | None |
| | 30.1 | None | None | None |
| | None | 34.4 | None | None |
| Chills | None | None | 3.3 | None |
| | None | None | None | 0.0 |
| | None | None | None | None |
| | 32.9 | None | None | None |
| | None | 57.4 | None | None |
| Headache | None | None | 25.6 | None |
| | None | None | None | 4.8 |
| | None | None | None | None |
| | 0.0 | None | None | None |
| | None | 1.6 | None | None |
| Rash | None | None | 1.1 | None |
| | None | None | None | 1.2 |
| | None | None | None | None |
| | 1.4 | None | None | None |
| | None | 8.2 | None | None |
| Others | None | None | 3.3 | None |
| | None | None | None | 0.0 |
| | None | None | None | None |
| | None | None | None | None |D. Solicited Systemic AEFIs for the Middle-aged Group
C. Solicited Local AEFIs for the Middle-aged Group
### Chart
| Category | Day 0 | Day 1 | Day 3 | Day 7 |
|---|---|---|---|---|
| | 38.3 | None | None | None |
| | None | 75.4 | None | None |
| Malaise | None | None | 12.5 | None |
| | None | None | None | 6.5 |
| | None | None | None | None |
| | 43.3 | None | None | None |
| | None | 87.7 | None | None |
| Fever | None | None | 4.2 | None |
| | None | None | None | 0.0 |
| | None | None | None | None |
| | 13.3 | None | None | None |
| | None | 26.3 | None | None |
| Fatigue | None | None | 8.3 | None |
| | None | None | None | 4.3 |
| | None | None | None | None |
| | 20.0 | None | None | None |
| | None | 33.3 | None | None |
| Myalgia | None | None | 4.2 | None |
| | None | None | None | 4.3 |
| | None | None | None | None |
| | 13.3 | None | None | None |
| | None | 47.4 | None | None |
| Arthralgia | None | None | 2.1 | None |
| | None | None | None | 0.0 |
| | None | None | None | None |
| | 5.0 | None | None | None |
| | None | 5.3 | None | None |
| Nausea/
Vomiting | None | None | 2.1 | None |
| | None | None | None | 0.0 |
| | None | None | None | None |
| | 0.0 | None | None | None |
| | None | 1.8 | None | None |
| Diarrhea | None | None | 2.1 | None |
| | None | None | None | 0.0 |
| | None | None | None | None |
| | 13.3 | None | None | None |
| | None | 31.6 | None | None |
| Chills | None | None | 2.1 | None |
| | None | None | None | 0.0 |
| | None | None | None | None |
| | 20.0 | None | None | None |
| | None | 47.4 | None | None |
| Headache | None | None | 18.8 | None |
| | None | None | None | 2.2 |
| | None | None | None | None |
| | 1.7 | None | None | None |
| | None | 1.8 | None | None |
| Rash | None | None | 0.0 | None |
| | None | None | None | 0.0 |
| | None | None | None | None |
| | 3.3 | None | None | None |
| | None | 5.3 | None | None |
| Others | None | None | 2.1 | None |
| | None | None | None | 0.0 |
| | None | None | None | None |
| | None | None | None | None |
### Chart
| Category | Day 0 | Day 1 | Day 3 | Day 7 |
|---|---|---|---|---|
| | 85.0 | None | None | None |
| | None | 93.0 | None | None |
| Injection-site pain | None | None | 37.5 | None |
| | None | None | None | 0.0 |
| | None | None | None | None |
| | 35.0 | None | None | None |
| | None | 52.6 | None | None |
| Localized warmth | None | None | 16.7 | None |
| | None | None | None | 0.0 |
| | None | None | None | None |
| | 1.7 | None | None | None |
| | None | 0.0 | None | None |
| Exanthema | None | None | 0.0 | None |
| | None | None | None | 0.0 |
| | None | None | None | None |
| | 6.7 | None | None | None |
| | None | 14.0 | None | None |
| Erythema | None | None | 20.8 | None |
| | None | None | None | 2.2 |
| | None | None | None | None |
| | 11.7 | None | None | None |
| | None | 21.1 | None | None |
| Swelling | None | None | 8.3 | None |
| | None | None | None | 2.2 |
| | None | None | None | None |
| | 8.3 | None | None | None |
| | None | 8.8 | None | None |
| Itchiness | None | None | 27.1 | None |
| | None | None | None | 8.7 |
| | None | None | None | None |
| | 0.0 | None | None | None |
| | None | 0.0 | None | None |
| Red eye | None | None | 0.0 | None |
| | None | None | None | 0.0 |
| | None | None | None | None |
| | 3.3 | None | None | None |
| | None | 8.8 | None | None |
| Others | None | None | 6.3 | None |
| | None | None | None | 4.3 |
| | None | None | None | None |

## Slide 6
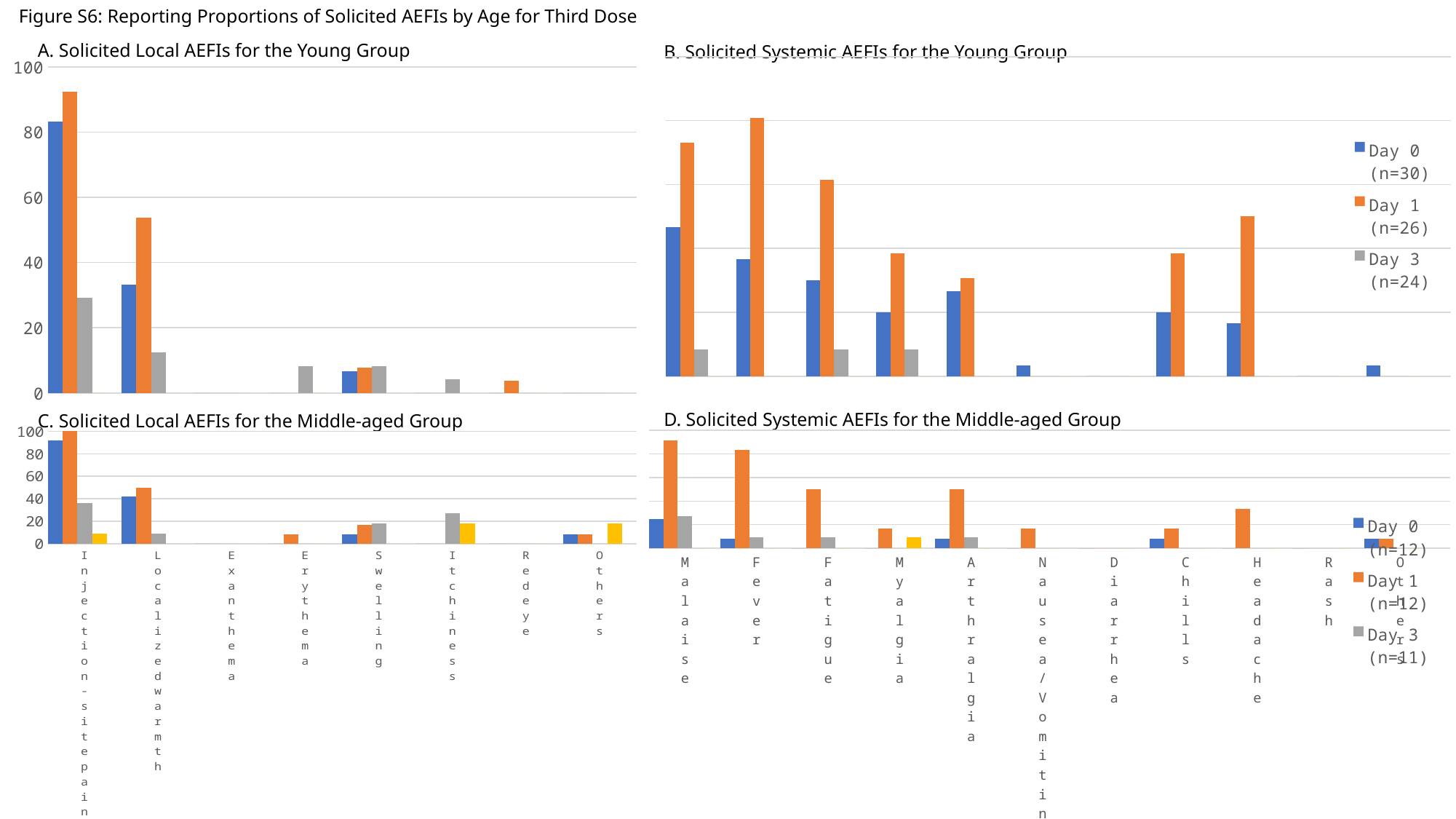

Figure S6: Reporting Proportions of Solicited AEFIs by Age for Third Dose
A. Solicited Local AEFIs for the Young Group
B. Solicited Systemic AEFIs for the Young Group
### Chart
| Category | Day 0 | Day 1 | Day 3 | Day 7 |
|---|---|---|---|---|
| | 83.3 | None | None | None |
| | None | 92.3 | None | None |
| Injection-site pain | None | None | 29.2 | None |
| | None | None | None | 0.0 |
| | None | None | None | None |
| | 33.3 | None | None | None |
| | None | 53.8 | None | None |
| Localized warmth | None | None | 12.5 | None |
| | None | None | None | 0.0 |
| | None | None | None | None |
| | 0.0 | None | None | None |
| | None | 0.0 | None | None |
| Exanthema | None | None | 0.0 | None |
| | None | None | None | 0.0 |
| | None | None | None | None |
| | 0.0 | None | None | None |
| | None | 0.0 | None | None |
| Erythema | None | None | 8.3 | None |
| | None | None | None | 0.0 |
| | None | None | None | None |
| | 6.7 | None | None | None |
| | None | 7.7 | None | None |
| Swelling | None | None | 8.3 | None |
| | None | None | None | 0.0 |
| | None | None | None | None |
| | 0.0 | None | None | None |
| | None | 0.0 | None | None |
| Itchiness | None | None | 4.2 | None |
| | None | None | None | 0.0 |
| | None | None | None | None |
| | 0.0 | None | None | None |
| | None | 3.8 | None | None |
| Red eye | None | None | 0.0 | None |
| | None | None | None | 0.0 |
| | None | None | None | None |
| | 0.0 | None | None | None |
| | None | 0.0 | None | None |
| Others | None | None | 0.0 | None |
| | None | None | None | 0.0 |
| | None | None | None | None |
### Chart
| Category | Day 0 | Day 1 | Day 3 | Day 7 |
|---|---|---|---|---|
| | 46.7 | None | None | None |
| | None | 73.1 | None | None |
| Malaise | None | None | 8.3 | None |
| | None | None | None | 0.0 |
| | None | None | None | None |
| | 36.7 | None | None | None |
| | None | 80.8 | None | None |
| Fever | None | None | 0.0 | None |
| | None | None | None | 0.0 |
| | None | None | None | None |
| | 30.0 | None | None | None |
| | None | 61.5 | None | None |
| Fatigue | None | None | 8.3 | None |
| | None | None | None | 0.0 |
| | None | None | None | None |
| | 20.0 | None | None | None |
| | None | 38.5 | None | None |
| Myalgia | None | None | 8.3 | None |
| | None | None | None | 0.0 |
| | None | None | None | None |
| | 26.7 | None | None | None |
| | None | 30.8 | None | None |
| Arthralgia | None | None | 0.0 | None |
| | None | None | None | 0.0 |
| | None | None | None | None |
| | 3.3 | None | None | None |
| | None | 0.0 | None | None |
| Nausea/
Vomiting | None | None | 0.0 | None |
| | None | None | None | 0.0 |
| | None | None | None | None |
| | 0.0 | None | None | None |
| | None | 0.0 | None | None |
| Diarrhea | None | None | 0.0 | None |
| | None | None | None | 0.0 |
| | None | None | None | None |
| | 20.0 | None | None | None |
| | None | 38.5 | None | None |
| Chills | None | None | 0.0 | None |
| | None | None | None | 0.0 |
| | None | None | None | None |
| | 16.7 | None | None | None |
| | None | 50.0 | None | None |
| Headache | None | None | 0.0 | None |
| | None | None | None | 0.0 |
| | None | None | None | None |
| | 0.0 | None | None | None |
| | None | 0.0 | None | None |
| Rash | None | None | 0.0 | None |
| | None | None | None | 0.0 |
| | None | None | None | None |
| | 3.3 | None | None | None |
| | None | 0.0 | None | None |
| Others | None | None | 0.0 | None |
| | None | None | None | 0.0 |
| | None | None | None | None |
| | None | None | None | None |D. Solicited Systemic AEFIs for the Middle-aged Group
C. Solicited Local AEFIs for the Middle-aged Group
### Chart
| Category | Day 0 | Day 1 | Day 3 | Day 7 |
|---|---|---|---|---|
| | 91.7 | None | None | None |
| | None | 100.0 | None | None |
| Injection-site pain | None | None | 36.4 | None |
| | None | None | None | 9.1 |
| | None | None | None | None |
| | 41.7 | None | None | None |
| | None | 50.0 | None | None |
| Localized warmth | None | None | 9.1 | None |
| | None | None | None | 0.0 |
| | None | None | None | None |
| | 0.0 | None | None | None |
| | None | 0.0 | None | None |
| Exanthema | None | None | 0.0 | None |
| | None | None | None | 0.0 |
| | None | None | None | None |
| | 0.0 | None | None | None |
| | None | 8.3 | None | None |
| Erythema | None | None | 0.0 | None |
| | None | None | None | 0.0 |
| | None | None | None | None |
| | 8.3 | None | None | None |
| | None | 16.7 | None | None |
| Swelling | None | None | 18.2 | None |
| | None | None | None | 0.0 |
| | None | None | None | None |
| | 0.0 | None | None | None |
| | None | 0.0 | None | None |
| Itchiness | None | None | 27.3 | None |
| | None | None | None | 18.2 |
| | None | None | None | None |
| | 0.0 | None | None | None |
| | None | 0.0 | None | None |
| Red eye | None | None | 0.0 | None |
| | None | None | None | 0.0 |
| | None | None | None | None |
| | 8.3 | None | None | None |
| | None | 8.3 | None | None |
| Others | None | None | 0.0 | None |
| | None | None | None | 18.2 |
| | None | None | None | None |
### Chart
| Category | Day 0 | Day 1 | Day 3 | Day 7 |
|---|---|---|---|---|
| | 25.0 | None | None | None |
| | None | 91.7 | None | None |
| Malaise | None | None | 27.3 | None |
| | None | None | None | 0.0 |
| | None | None | None | None |
| | 8.3 | None | None | None |
| | None | 83.3 | None | None |
| Fever | None | None | 9.1 | None |
| | None | None | None | 0.0 |
| | None | None | None | None |
| | 0.0 | None | None | None |
| | None | 50.0 | None | None |
| Fatigue | None | None | 9.1 | None |
| | None | None | None | 0.0 |
| | None | None | None | None |
| | 0.0 | None | None | None |
| | None | 16.7 | None | None |
| Myalgia | None | None | 0.0 | None |
| | None | None | None | 9.1 |
| | None | None | None | None |
| | 8.3 | None | None | None |
| | None | 50.0 | None | None |
| Arthralgia | None | None | 9.1 | None |
| | None | None | None | 0.0 |
| | None | None | None | None |
| | 0.0 | None | None | None |
| | None | 16.7 | None | None |
| Nausea/
Vomiting | None | None | 0.0 | None |
| | None | None | None | 0.0 |
| | None | None | None | None |
| | 0.0 | None | None | None |
| | None | 0.0 | None | None |
| Diarrhea | None | None | 0.0 | None |
| | None | None | None | 0.0 |
| | None | None | None | None |
| | 8.3 | None | None | None |
| | None | 16.7 | None | None |
| Chills | None | None | 0.0 | None |
| | None | None | None | 0.0 |
| | None | None | None | None |
| | 0.0 | None | None | None |
| | None | 33.3 | None | None |
| Headache | None | None | 0.0 | None |
| | None | None | None | 0.0 |
| | None | None | None | None |
| | 0.0 | None | None | None |
| | None | 0.0 | None | None |
| Rash | None | None | 0.0 | None |
| | None | None | None | 0.0 |
| | None | None | None | None |
| | 8.3 | None | None | None |
| | None | 8.3 | None | None |
| Others | None | None | 0.0 | None |
| | None | None | None | 0.0 |
| | None | None | None | None |
| | None | None | None | None |
